# Supplementary material for: Face masks while exercising trial (MERIT): a cross-over randomised controlled study
Source: BMJ Open. 2023 Jan 5;13(1):e063014. doi: 10.1136/bmjopen-2022-063014 (PMC9827243; doi:10.1136/bmjopen-2022-063014)
Supplement: Supplementary data [file bmjopen-2022-063014supp002.pdf]

Face masks and exercise (c29.9.20)

Page 1: Face masks and oxygen levels - what happens during exercise?

Introduction

Thank you for completing your exercise session, wearing the different face masks. We hope that you are now ready to upload your results. This should take around 10 minutes.

There are no right or wrong answers to any of the questions. Please be as honest as possible when answering. Your answers will be confidential and will not be shared with anyone other than the research team. We will not be collecting any identifiable information at this stage to protect your privacy.

By completing this questionnaire, you are consenting to your anonymous information being used for research on the impact of wearing a face mask whilst exercising.

Thank you again for supporting this research.

If you have any questions, please contact:

Dr Nicholas Jones (nicholas.jones2@phc.ox.ac.uk)

Jones N, *et al. BMJ Open* 2023; 13:e063014. doi: 10.1136/bmjopen-2022-063014

Page 2: Basic details

1. How old are you?

2. Which gender do you identify with?

3. How many days do you usually exercise per week?

4. Do you smoke?

4.a. If you smoke, how many cigarettes a day do you smoke on average?

5. What type of exercise did you do?

Jones N, *et al. BMJ Open* 2023; 13:e063014. doi: 10.1136/bmjopen-2022-063014

6. When did you complete the exercise session?

Please enter a date and time in the format 'DD/MM/YYYY HH:MM', for example 27/03/1980 15:43.

(dd/mm/yyyy hh:mm)

COVID screening questions

7. Where did you complete the study session?

- London
- Oxford
- Nottingham

8. Please confirm you do not have any symptoms of possible COVID-19 on the day of the study session

- No symptoms
- I have a fever
- I have a cough
- I have a change in sense of smell or taste

9. To the best of your knowledge, are other members of your household free from symptoms of COVID-19?

Jones N, *et al. BMJ Open* 2023; 13:e063014. doi: 10.1136/bmjopen-2022-063014

- Yes
- No

IF YOU OR SOMEONE YOU LIVE WITH HAVE ANY SYMPTOMS OF COVID-19, YOU MUST NOT TAKE PART IN THE STUDY TODAY. PLEASE LET A MEMBER OF THE STUDY TEAM KNOW AS SOON AS POSSIBLE.

Page 3: Oxygen saturations

The following questions will require you to input the oxygen saturation levels you recorded at different time points during each of the four exercise periods. Please also add your oxygen saturations at the start of exercise / resting oxygen saturations below.

10. What are your resting oxygen saturation levels?

11. Please record your oxygen saturations during exercise at each of the following time points. You should input an answer into every box.

	Oxygen levels 7 and a half minutes into exercise	Oxygen levels at the end of exercise	Oxygen levels 1 minute after ending exercise
No face mask	Please select	Please select	Please select
Cloth face mask	Please select	Please select	Please select
Surgical face mask	Please select	Please select	Please select
FFR (respirator) face mask	Please select	Please select	Please select

Jones N, *et al. BMJ Open* 2023; 13:e063014. doi: 10.1136/bmjopen-2022-063014

Page 4: Heart rate and exercise

The following questions will require you to input your heart rate levels, which you recorded at different time points during each of the four exercise periods. Please also add your heart rate at the start of exercise / resting heart rate below.

12. What is your resting heart rate?

13. Please record your heart rate during exercise at each of the following time points. You should input an answer into every box.

	Heart rate 7 and a half minutes into exercise	Heart rate at the end of exercise	Heart rate 1 minute after ending exercise
No face mask	Please select	Please select	Please select
Cloth face mask	Please select	Please select	Please select
Surgical face mask	Please select	Please select	Please select
FFR (respirator) face mask	Please select	Please select	Please select

Jones N, *et al. BMJ Open* 2023; 13:e063014. doi: 10.1136/bmjopen-2022-063014

Page 5: Distance travelled

Please record data here regarding the distance you travelled during exercise, including average speed data if available (please provide units).

14. Distance travelled exercising wearing **no face mask**

15. Average speed exercising with **no face mask**

16. Distance travelled exercising wearing a **cloth face mask**

17. Average speed exercising wearing a **cloth face mask**

18. Distance travelled exercising wearing a **surgical face mask**

Jones N, *et al. BMJ Open* 2023; 13:e063014. doi: 10.1136/bmjopen-2022-063014

19. Average speed exercising wearing a **surgical face mask**

20. Distance travelled exercising wearing a **FFR (respirator) face mask**

21. Average speed exercising wearing a FFR (respirator) face mask

Jones N, *et al. BMJ Open* 2023; 13:e063014. doi: 10.1136/bmjopen-2022-063014

Page 6: Comfort of wearing a face mask

22. How comfortable was the mask (or no mask) during this exercise session? (Rate from 1 to 9, 1= extremely comfortable, 9= extremely uncomfortable)

	Comfort level
No face mask	Please select
Cloth face mask	Please select
Surgical face mask	Please select
FFR (respirator) face mask	Please select

22.a. Were you able to complete all four exercises sessions? If not please state which you did not complete and why.

23. How easy did you find it to exercise during each session? (Rate from 0 to 10; 0=rest, 1=really easy, 2=easy, 3=moderate, 4=sort of hard, 5=hard, 7= really hard, 9= really, really hard, 10= maximal) [Borg Scale]

	Ease of exercise										
	0	1	2	3	4	5	6	7	8	9	10
No face mask	☐	☐	☐	☐	☐	☐	☐	☐	☐	☐	☐
Cloth face mask	☐	☐	☐	☐	☐	☐	☐	☐	☐	☐	☐
Surgical face mask	☐	☐	☐	☐	☐	☐	☐	☐	☐	☐	☐
FFR (respirator) mask	☐	☐	☐	☐	☐	☐	☐	☐	☐	☐	☐

24. Which face mask did you find most difficult to exercise wearing?

Jones N, *et al. BMJ Open* 2023; 13:e063014. doi: 10.1136/bmjopen-2022-063014

Please don't select more than 1 answer(s) per row.

	Cloth mask	Surgical mask	FFR (respirator) mask
Please select one	☐	☐	☐

25. How easy was it to breath during each exercise session? Please rate from 1 to 9, 1 = extremely easy, 9 = extremely difficult)

	1	2	3	4	5	6	7	8	9
No face mask	☐	☐	☐	☐	☐	☐	☐	☐	☐
Cloth face mask	☐	☐	☐	☐	☐	☐	☐	☐	☐
Surgical face mask	☐	☐	☐	☐	☐	☐	☐	☐	☐
FFR (respirator) face mask	☐	☐	☐	☐	☐	☐	☐	☐	☐

25.a. Please add any relevant comments you have on the ease of exercising wearing a face mask

26. Did you feel any other symptoms during exercise that were different to usual?

- ☐ Headache
- ☐ More breathless
- ☐ Claustrophobia
- ☐ Anxiety
- ☐ Fatigued
- ☐ Drowsy
- ☐ Dizzy
- ☐ Other

26.a. If other please specify

27. Overall, how would you feel about being required to exercise wearing a face mask to limit the spread of COVID-19?

Jones N, *et al. BMJ Open* 2023; 13:e063014. doi: 10.1136/bmjopen-2022-063014
