# Supplementary material for: Face masks while exercising trial (MERIT): a cross-over randomised controlled study
Source: BMJ Open. 2023 Jan 5;13(1):e063014. doi: 10.1136/bmjopen-2022-063014 (PMC9827243; doi:10.1136/bmjopen-2022-063014)
Supplement: Supplementary data [file bmjopen-2022-063014supp001.pdf]

BMJ Publishing Group Limited (BMJ) disclaims all liability and responsibility arising from any reliance placed on this supplemental material which has been supplied by the author(s)

Supplemental Table 1a. Changes in oxygen saturations comparing different mask types worn during exercise. Sub-group analysis of participants with resting oxygen saturations of ≤ 98%

Comparison	Average oxygen saturations (%) with mask	Average oxygen saturations (%), no mask	Estimated average difference (%, 95% confidence interval)	Non-inferior @ -2%
During exercise (N = 30)				
Cloth - None	96.5	97.4	-0.90 (-1.48 to -0.32)	TRUE
Surgical - None	97.3	97.4	-0.12 (-0.72 to 0.49)	TRUE
FFP3 - None	96.5	97.4	-0.88 (-1.47 to -0.29)	TRUE
End of exercise (N = 29)				
Cloth - None	96.9	97.0	-0.07 (-0.57 to 0.43)	TRUE
Surgical - None	97.0	97.0	-0.03 (-0.55 to 0.49)	TRUE
FFP3 - None	97.0	97.0	0.00 (-0.52 to 0.52)	TRUE
1 minute after the end of exercise (N = 30)				
Cloth - None	97.6	97.7	-0.11 (-0.61 to 0.39)	TRUE
Surgical - None	97.9	97.7	0.18 (-0.32 to 0.67)	TRUE
FFP3 - None	97.6	97.7	-0.08 (-0.57 to 0.42)	TRUE

Supplemental Table 1b. Changes in oxygen saturations comparing different mask types worn during exercise. Sub-group analysis of participants with resting oxygen saturations of > 98%

Comparison	Average oxygen saturations (%) with mask	Average oxygen saturations (%), no mask	Estimated average difference (%, 95% confidence interval)	Non-inferior @ -2%
During exercise (N = 39)				
Cloth - None	97.3	97.9	-0.69 (-1.11 to -0.27)	TRUE
Surgical - None	97.5	97.9	-0.40 (-0.83 to 0.03)	TRUE
FFP3 - None	97.2	97.9	-0.77 (-1.19 to -0.35)	TRUE
End of exercise (N = 39)				
Cloth - None	97.2	97.2	-0.07 (-0.48 to 0.35)	TRUE
Surgical - None	97.7	97.2	0.51 (0.11 to 0.92)	TRUE
FFP3 - None	96.9	97.2	-0.36 (-0.76 to 0.05)	TRUE
1 minute after the end of exercise (N = 37)				
Cloth - None	97.8	97.7	0.16 (-0.18 to 0.49)	TRUE
Surgical - None	98.2	97.7	0.46 (0.13 to 0.79)	TRUE
FFP3 - None	98.1	97.7	0.32 (-0.01 to 0.66)	TRUE

Supplemental Table 2a. Changes in heart rate comparing different mask types worn during exercise. Sub-group analysis of participants with resting oxygen saturations of ≤ 98%

Contrast	Average heart rate (%) with mask (beats per minute)	Average heart rate (%), no mask (beats per minute)	Estimated average difference (bpm, 95% confidence interval)	Non-inferior @ +7bpm
During exercise (N = 29)				
Cloth - None	156.4	158.0	-1.69 (-6.55 to 3.17)	TRUE
Surgical - None	155.9	158.0	-1.41 (-6.57 to 3.75)	TRUE
FFP3 - None	159.5	158.0	2.21 (-2.95 to 7.37)	FALSE
End of exercise (N = 29)				
Cloth - None	151.5	155.9	-4.17 (-10.89 to 2.54)	TRUE
Surgical - None	156.5	155.9	0.36 (-6.59 to 7.31)	FALSE
FFP3 - None	158.4	155.9	2.04 (-4.91 to 8.99)	FALSE
1 minute after the end of exercise (N = 29)				
Cloth - None	123.5	123.2	0.15 (-4.47 to 4.77)	TRUE
Surgical - None	125.4	123.2	2.74 (-1.88 to 7.36)	FALSE
FFP3 - None	125.7	123.2	2.82 (-1.96 to 7.60)	FALSE

Supplemental Table 2b. Changes in heart rate comparing different mask types worn during exercise. Sub-group analysis of participants with resting oxygen saturations of > 98%

Contrast	Average heart rate (%) with mask (beats per minute)	Average heart rate (%), no mask (beats per minute)	Estimated average difference (bpm, 95% confidence interval)	Non-inferior @ +7bpm
During exercise (N = 39)				
Cloth - None	158.4	156.8	1.59 (-2.22 to 5.40)	TRUE
Surgical - None	160.1	156.8	3.39 (-0.49 to 7.27)	FALSE
FFP3 - None	161.6	156.8	4.74 (0.93 to 8.55)	FALSE
End of exercise (N = 39)				
Cloth - None	162.2	159.9	2.11 (-1.03 to 5.26)	TRUE
Surgical - None	160.5	159.9	0.54 (-2.54 to 3.62)	TRUE
FFP3 - None	159.8	159.9	-0.18 (-3.26 to 2.90)	TRUE
1 minute after the end of exercise (N = 37)				
Cloth - None	126.2	125.3	0.71 (-2.31 to 3.73)	TRUE
Surgical - None	124.5	125.3	-0.97 (-3.93 to 1.99)	TRUE
FFP3 - None	125.7	125.3	0.73 (-2.23 to 3.69)	TRUE

Supplemental Table 3a. Changes in oxygen saturations comparing different mask types worn during exercise. Sensitivity analysis excluding the one participant who completed the exercise rowing instead of running

Comparison	Average oxygen saturations (%) with mask	Average oxygen saturations (%), no mask	Estimated average difference (%, 95% confidence interval)	Non-inferior @ -2%
During exercise (N = 68)				
Cloth - None	97.0	97.7	-0.75 (-1.09 to -0.41)	TRUE
Surgical - None	97.5	97.7	-0.27 (-0.63 to 0.08)	TRUE
FFP3 - None	96.9	97.7	-0.81 (-1.16 to -0.47)	TRUE
End of exercise (N = 67)				
Cloth - None	97.1	97.1	-0.05 (-0.38 to 0.27)	TRUE
Surgical - None	97.4	97.1	0.30 (-0.02 to 0.63)	TRUE
FFP3 - None	96.9	97.1	-0.21 (-0.54 to 0.11)	TRUE
1 minute after the end of exercise (N = 66)				
Cloth - None	97.7	97.7	0.05 (-0.24 to 0.34)	TRUE
Surgical - None	98.1	97.7	0.35 (0.07 to 0.64)	TRUE
FFP3 - None	97.9	97.7	0.16 (-0.12 to 0.45)	TRUE

Supplemental Table 3b. Changes in heart rate comparing different mask types worn during exercise. Sensitivity analysis excluding the one participant who completed the exercise rowing instead of running

Contrast	Average heart rate (%) with mask (beats per minute)	Average heart rate (%), no mask (beats per minute)	Estimated average difference (bpm, 95% confidence interval)	Non-inferior @ +7bpm
During exercise (N = 68)				
Cloth - None	157.6	157.4	0.09 (-2.95 to 3.13)	TRUE
Surgical - None	157.8	157.4	0.67 (-2.49 to 3.83)	FALSE
FFP3 - None	160.0	157.4	2.85 (-0.27 to 5.97)	FALSE
End of exercise (N = 68)				
Cloth - None	157.0	158.6	-1.58 (-4.98 to 1.83)	TRUE
Surgical - None	158.8	158.6	0.07 (-3.35 to 3.49)	TRUE
FFP3 - None	159.1	158.6	0.22 (-3.20 to 3.64)	TRUE
1 minute after the end of exercise (N = 66)				
Cloth - None	124.9	124.3	0.50 (-2.07 to 3.07)	TRUE
Surgical - None	124.7	124.3	0.55 (-1.99 to 3.09)	TRUE
FFP3 - None	125.7	124.3	1.81 (-0.77 to 4.39)	TRUE

Supplemental table 4. Participants free text comments regarding the ease of exercising wearing different mask types. These have been categorised as either positive, negative or neutral comments and by facemask type.

Positive comments	Neutral comments	Negative comments
**Any mask**		
It wasn't actually that much worse than exercising with no mask and it did keep my face warm on a very cold day.	Inconvenient but bearable	Considerably more difficult than normal
Didn't find that any mask slowed me down.	As the exercise went on it became easier to breathe with a mask on	With all of them I didn't actually become short of breath it was more discomfort. With the cloth one and the surgical one slightly the main issue was due to them being flexible the fabric would go into my mouth when breathing in.
	It is relevant whether the mask is stable	Masks get in the way, maybe not on the actual performance. Placebo effect
	It was slightly more uncomfortable than not wearing a mask, but not too bad.	All of the masks made my face extra sweaty
	All were warmer than no mask	I'd prefer not to exercise with a mask. There was a lot of humidity with the tighter fitting masks and it makes you much more aware of your breathing.
		The buff and surgical masks were similar - not too bad but a lot worse than no mask. It was harder across the 15 minutes with condensation from the mask. You feel you overheat and there is steaming up of your glasses
		The humidity was unpleasant rather than being difficult to breathe. I found it harder to breathe through my mouth wearing any of the masks.
		It wasn't that much more difficult wearing a mask but the sweatiness was unpleasant
		Surprised to cover roughly the same distance each time even when it more difficult in some of the masks than others - the cloth and FFR.
		the FFP and cloth mask were much worse
**Cloth**		
Cloth was good for me as I wear glasses and allowed me to breathe through my nose and mouth more easily		Cloth mask was surprisingly uncomfortable - about as bad as wearing the FFP2 mask. Noticed that the cloth mask quickly became soaked through with sweat and water vapour which made it very difficult to breathe through the nose.

The cloth mask was particularly uncomfortable as it clung to my face and was very distracting every time you breathe. This was a particular problem as I was breathing through my mouth during the exercises

None were great. The cloth had the added disadvantage of making the rest of your face hot

Cloth mask gets very moist and gets inhaled and falls a lot. Breathing pattern altered with masks - not breathing in, out, in, out but more in, in, out, in, in, out...

Cloth mask felt quite claustrophobic

Cloth mask tight and hot around face

Cloth mask got wet

Cloth mask is distracting (slips a lot).

It was hard to breathe through my mouth with the cloth mask

Wearing cloth mask was hardest because the cloth became humid and block air from entering and exiting mouth.

Cloth mask is the worst to wear when I do exercise

Cloth mask so very hard to breathe in

The cloth mask was annoying because it kept slipping down.

Cloth face mask kept falling down

Cloth mask kept going in my mouth

Running with the mask fogged up my glass, which was annoying. This was worst with the cloth mask

The buff cloth masked pressed down on my nose, which meant I had to breathe through my nose (not a good thing).

Dont make people wear a cloth mask, it sucks in when breathing and it's difficult to breathe.

The cloth mask kept falling and was terrible.

The buff mask was worse as it would suck into your mouth.

I was surprised that I found the cloth mask harder

Cloth was uncomfortable due to the stickiness

Cloth mask felt very close and humid.

Cloth mask was the worst, got very wet, like being waterboarded, sweaty, incompatible with glasses.

		Cloth mask kept slipping down and being sucked in when breathing in
		Didn't like cloth mask - too hot, too close to mouth, claustrophobic
		Buff was generally horrible
		Cloth mask didn't fit well with glasses, kept slipping. Cloth mask was very sticky, stuck straight to nose and mouth, really uncomfortable, no gap to breathe.
		Cloth mask kept getting in my mouth, couldn't out of nose well.
		Cloth mask kept going in my mouth.
		FFR better than cloth more room to breathe
		The buff was claustrophobic
		Cloth mask was hardest, kept sliding down, and going in to mouth when breathing.
		Cloth kept sliding down and made face hot but otherwise didn't mind.
		Cloth gets in mouth when breathing in, kept having to pull it up.
**FFP3**		
I was surprised the FFR was easier than the others - it helped that it doesn't move around much	Exercising in the FFR wasn't as bad as I thought it'd be	The FFR was extremely difficult to exercise with compared to the rest - I was out of breath constantly and I had to slow down or switch to walking just to catch my breath. While wearing the FFR mask, I also felt a lot of strain (pressure) in my eyes.
FFR was very comfortable		Got nose pain from metal on resp mask
The FFR was a good fit		FFR mask is constraining.
Found FFR similar to the surgical mask - both were fine		It was hard to breathe through my nose with the FFR mask.
Liked the FFP - less annoying, better than expected, didn't move, didn't interfere with glasses.		Felt dizzy with FFR mask
FFR easiest to breathe in		The FFR was definitely the worst, it was so tight fitting it felt difficult to breathe
Found FFR to be the same as normal		The FFR mask was very tight fitting and it felt as though my heart rate was going very high when I was wearing it.
FFR didn't shake at all whilst running unlike the others. Didn't really notice I was wearing the FFR mask, although got quite		It felt like the FFR restricted your breathing

warm		
FFR was amenable to glasses-wearing, the fixed gap made it easier to breathe, found it fine in general.		FFR worse than surgical.
I felt close to normal in the FFP		FFR wasn't as bad as I thought it'd be, although still quite bad and the worse one for me. I found the shape around the nose made it more difficult to breathe
		Felt really lightheaded and struggled to breathe when wearing FFR, really uncomfortable.
		felt a bit dizzy wearing FFR
		FFR sweaty, made face really hot, but was FFR was easier to breathe in compared to cloth
		FFR - nose trapped, couldn't breathe as easily, bad smell to mask.
		FFR - tight around nose, hard to breathe at times.
**Surgical**		
The surgical mask was fine breathing	Cloth mask was ok, surgical was slightly better.	Surgical mask moves around a lot and does not feel very helpful in filtering air supply.
I wouldn't choose to wear a mask but the surgical mask was definitely best.	Surgical was fine but sweaty.	The surgical mask accumulated a lot of water vapour and steamed my glasses.
The surgical mask was best- I could run with that.	Surgical mask was ok	Surgical mask moved around a lot on my face, uncomfortable.
Found FFR similar to the surgical mask - both were fine	Hospital mask was fine, bearable.	Didn't like surgical - steamed up glasses
No mask and surgical felt the same		
Surgical mask wasn't too bad, worked better with glasses than FFR.		
Found surgical mask fine.		
Surgical mask felt same as no mask don't really notice you're wearing it, although may think differently in the summer.		
There was little restriction with the surgical mask		
Hospital was best.		

BMJ Publishing Group Limited (BMJ) disclaims all liability and responsibility arising from any reliance placed on this supplemental material which has been supplied by the author(s)

Surgical mask was fine, easiest one.

Supplemental table 5. Participants written, free-text responses to the question, ‘Overall, how would you feel about being required to exercise wearing a face mask to limit the spread of COVID-19?’ These were categorised as being either supportive, against or neutral.

**Supportive**	**Against**	**Neutral**
If it was in the interests of public health and safety, I would have no issues wearing a mask to limit the spread of COVID-19. Of the masks worn, my strong preference would be for the FFP3 mask as it was by far the most comfortable	Do not feel it's worth the hassle	Was unpleasant exercising with any of the face coverings on but if I had to pick one to wear it would definitely be the surgical mask.
Would wear surgical mask if it was necessary	Not great. I would wear the surgical mask though	Mixed, the best mask was the respirator one but those are expensive to get. However, as an issue of public safety I wouldn't have a problem wearing one if told it was the best thing to do.
It would make no real impact to my exercise having to wear a face mask or not	Negative	It would depends on what type of mask I had. I would probably look into perhaps a running mask if such a thing exists. Failing that maybe a surgical one but it would certainly limit the exercise I would want to do as it is noticeably harder and less enjoyable to at least run in a mask.
It would be fine if it is moderate intensity exercise. If I was pushing extremely hard it may slightly limit me. If I was in a poorly ventilated gym I would feel more comfortable wearing a face mask	At least for exercise that significantly increase oxygen requirement, I would find it very challenging and would try to avoid	Not keen but would do it
Fine with it	I think its a really bad idea to have to wear a mask while exercising as it can make you very out of breath and I would fear it could even be fatal in some circumstances.	Fine but not ideal
Only would wear surgical mask	Wouldn't exercise	Wouldn’t mind if had to but would prefer not to
I would be happy to but it’s a lot less enjoyable	Would rather have a choice in the matter	Outside, I would not like it but it would be more pleasant as the wind is blowing on the rest of your face reducing the heat. Inside I would not like it due to the heat. It would definitely put me off exercising.
Fine if indoors and high risk setting but not so keen outdoors in large open spaces.	I would feel annoyed because outside its useless. It would make sense just inside	Surgical face masks would be a bit inconvenient but definitely an option especially in a gym context

I would be ok to exercise with the surgical mask, but none of the other ones	Would not support it at all. 2 out of 10	I would do what is necessary to protect others. However, with current evidence pointing towards the conclusion that isolated outdoors exercise would not effect COVID spread like eating in a pub would, I'm not inclined to wear one.
I think it is important to have a mask on when running in crowded public areas, otherwise it should not be required.	I believe we don't need to wear any face mask outdoors	If indoors - makes sense. Outdoors - a bit silly for me
Fine, just takes a while to adjust to wearing a face mask	I would still exercise but I wouldn't be happy. I'd also be annoyed because I don't think that it's really needed for preventing transmission, especially outdoors.	I would if I had to.
I would and have only used a surgical mask when required to wear one	I'd rather not unless I had to	I would rather not though if it was a rule I would do it to continue to exercise.
It is OK for me to wear a surgical mask (during exercise)	I would prefer not to	I'd wear the surgical mask if I had to wear something.
I agree to wear a face mask to do the exercise for safety, and the surgical mask did not interfere with my breathing	I'd prefer not to. If I had to I'd wear the surgical mask.	I would if needed to inside, don't see the point of having to wear one whilst exercising outside
I wouldnt mind if it was the surgical mask but it would definitely make me go slower	I wouldn't be happy with this, wearing a mask to exercise is uncomfortable and sweaty. When you're wearing one to exercise outdoor people give you looks, no one wears one outdoors	Would it have to, would choose the FFP
Exercising wearing a mask would be OK if we had to	Would prefer not to, if had to would wear FFR as didn't make glasses steam up	I wouldnt like to but would wear the surgical mask if we had to
Happy to wear the surgical face mask, but not the FFR or Cloth face mask	Would rather not, but would if really had to. Would choose to wear surgical.	I would tolerate it but not advocate it.
Wouldn't mind having to wear the surgical mask	Would rather not, would choose to wear surgical mask if had to, if forced to wear the others (cloth or FFR) I would choose not to exercise	
Felt it was possible to exercise in all 3, but less comfortable in FFR	Would prefer not to, wouldn't mind wearing surgical or cloth although not	

	ideal. Wouldn't wear FFR
I'd be happy to do it in an indoor gym if required to do so.	Wouldn't want to, hated it
I would be okay with it	Wouldn't do it, would just go outside to exercise instead. Would consider hospital mask as that was the best.
Only if it was the surgical mask	I wouldn't be happy. When I ran with no mask at the end it felt like breathing in HD
I would be fine with that.	
I would be happy to wear the surgical mask	
Having done so for the study, I wouldn't mind.	
Would choose to wear the surgical mask.	
Wouldn't mind doing so indoors, wouldn't mind wearing surgical or FFR	
Wouldn't mind doing so indoors, think it'd be unnecessary outdoors	
Wouldn't bother me too much, would choose to wear surgical.	
Not sure how they'd be in more intense exercise or hotter conditions. Would be fine on a run but more difficult if required to communicate in team sports e.g. football	
Would wear surgical mask, possibly buff for short distances were cardio is concerned	
Overall it was close to normal exercising wearing a mask. I would feel okay about doing this if it was not the cloth mask.	
To be honest if wearing a face mask during exercise will limit the spread of Covid then I am happy to do so	
