# Supplementary material for: Face masks while exercising trial (MERIT): a cross-over randomised controlled study
Source: BMJ Open. 2023 Jan 5;13(1):e063014. doi: 10.1136/bmjopen-2022-063014 (PMC9827243; doi:10.1136/bmjopen-2022-063014)
Supplement: Author's manuscript [file bmjopen-2022-063014.draft_revisions.pdf]

BMJ Open is committed to open peer review. As part of this commitment we make the peer review history of every article we publish publicly available.

When an article is published we post the peer reviewers' comments and the authors' responses online. We also post the versions of the paper that were used during peer review. These are the versions that the peer review comments apply to.

The versions of the paper that follow are the versions that were submitted during the peer review process. They are not the versions of record or the final published versions. They should not be cited or distributed as the published version of this manuscript.

BMJ Open is an open access journal and the full, final, typeset and author-corrected version of record of the manuscript is available on our site with no access controls, subscription charges or pay-per-view fees (http://bmjopen.bmj.com).

If you have any questions on BMJ Open's open peer review process please email info.bmjopen@bmj.com

BMJ Open

FaceMasks whilst ExeRcIsing Trial (MERIT): a crossover randomised controlled study

Journal:	*BMJ Open*
Manuscript ID	bmjopen-2022-063014
Article Type:	Original research
Date Submitted by the Author:	22-Mar-2022
Complete List of Authors:	Jones, Nicholas; University of Oxford, Primary Care Health Sciences<br>Oke, Jason; University of Oxford, Nuffiled Department of Primary Care Health Sciences<br>Marsh, Seren; University of Oxford<br>Nikbin, Kurosh; Kings College London<br>Bowley, Jonathan; University of Nottingham<br>Dijkstra, H Paul ; University of Oxford, Department of Continuing Education ; Aspetar Orthopaedic and Sports Medicine Hospital, Medical Education Department<br>Hobbs, Richard; University of Oxford, Dept of Primary Health Care<br>Greenhalgh, Trisha; University of Oxford, Nuffield Department of Primary Care Health Sciences
Keywords:	COVID-19, Public health < INFECTIOUS DISEASES, PREVENTIVE MEDICINE

SCHOLARONE™
Manuscripts

I, the Submitting Author has the right to grant and does grant on behalf of all authors of the Work (as defined in the below author licence), an exclusive licence and/or a non-exclusive licence for contributions from authors who are: i) UK Crown employees; ii) where BMJ has agreed a CC-BY licence shall apply, and/or iii) in accordance with the terms applicable for US Federal Government officers or employees acting as part of their official duties; on a worldwide, perpetual, irrevocable, royalty-free basis to BMJ Publishing Group Ltd ("BMJ") its licensees and where the relevant Journal is co-owned by BMJ to the co-owners of the Journal, to publish the Work in this journal and any other BMJ products and to exploit all rights, as set out in our licence.

The Submitting Author accepts and understands that any supply made under these terms is made by BMJ to the Submitting Author unless you are acting as an employee on behalf of your employer or a postgraduate student of an affiliated institution which is paying any applicable article publishing charge ("APC") for Open Access articles. Where the Submitting Author wishes to make the Work available on an Open Access basis (and intends to pay the relevant APC), the terms of reuse of such Open Access shall be governed by a Creative Commons licence – details of these licences and which Creative Commons licence will apply to this Work are set out in our licence referred to above.

Other than as permitted in any relevant BMJ Author's Self Archiving Policies, I confirm this Work has not been accepted for publication elsewhere, is not being considered for publication elsewhere and does not duplicate material already published. I confirm all authors consent to publication of this Work and authorise the granting of this licence.

For peer review only

FaceMasks whilst ExeRcIsing Trial (MERIT): a crossover randomised controlled study

[1]Nicholas R Jones

[1]Jason L. Oke

[2]Seren Marsh

[3]Kurosh Nikbin

[4]Jonathan Bowley

[5,6]H Paul Dijkstra

[1]FD Richard Hobbs

[1]Trisha Greenhalgh

1. Nuffield Department of Primary Care Health Sciences, University of Oxford, Oxford, UK
2. University of Oxford Medical School, University of Oxford, Oxford, UK
3. GKT School of Medical Education, Kings College London, London, UK
4. School of Medicine, University of Nottingham, Nottingham, UK
5. Medical Education Department, Aspetar Orthopaedic and Sports Medicine Hospital, Doha, Qatar
6. Department for Continuing Education, University of Oxford, Oxford, UK

Corresponding author: Dr Nicholas Jones, Nuffield Department of Primary Care Health Sciences, University of Oxford, Radcliffe Primary Care Building, Woodstock Road, Oxford OX2 6GG.

Email: nicholas.jones2@phc.ox.ac.uk. Twitter @drnrjones. ORCID ID 0000-0002-0352-3785

Word count: 3022

Abstract word count: 286

For peer review only - http://bmjopen.bmj.com/site/about/guidelines.xhtml

ABSTRACT

Objectives: Physical exertion is a high-risk activity for aerosol emission of respiratory pathogens, but there is controversy around whether facemasks are safe and acceptable when exercising. We aimed to determine the safety and tolerability of healthy young adults wearing different types of facemask during moderate-to-high intensity exercise.

Design: Crossover randomised controlled study.

Participants: We recruited people aged 18 to 35 years, who exercised at least three times per week and had no pre-existing health condition that limited their ability to exercise. All 72 volunteers (mean age 23.1 years) completed the study.

Interventions: Comparison of wearing a surgical, cloth and FFP3 mask to no mask during 15 minutes of exercise separated by 5 minutes rest. Each participant completed each arm in random order.

Primary outcome measure: In a non-inferiority analysis, the primary outcome was changes in oxygen saturations (non-inferiority margin=2%).

Results: Changes in oximeter readings did not exceed the pre-specified non-inferiority margin with any mask type compared to no mask. At the end of exercise the estimated average difference in oxygen saturations for cloth mask was -0.07% (95%CI -0.39 to 0.25), for surgical 0.28% (-0.04 to 0.60) and for FFP3 -0.21% (-0.53 to 0.11). The corresponding estimated average difference in heart rate for cloth mask was -1.20 bpm (-4.56 to 2.15), for surgical 0.36 bpm (-3.01 to 3.73) and for FFP3 0.52 bpm (-2.85 to 3.89). Wearing a facemask caused additional symptoms such as breathlessness (n=13, 18) and dizziness (n=7, 10%). 33 participants broadly supported facemask wearing during exercise, particularly indoors, but 22 were opposed.

Conclusion: This study adds to previous findings (mostly from non-randomised studies) that exercising at moderate-to-high intensity wearing a facemask appears to be safe in healthy, young adults.

Trial registration: clinicaltrials.gov, identifier NCT04932226

For peer review only - http://bmjopen.bmj.com/site/about/guidelines.xhtml

Strengths and limitations of this study

- This is the largest study to date assessing the safety and tolerability of exercising wearing a facemask.
- The randomised crossover design ensured that the effect of any fatigue from exercise was equal between mask types.
- Participants exercised outdoors and during cold weather, pulse oximeters were sometimes slow to provide readings, which may have resulted in a recovery in oxygen levels and an under-estimate of effect size.
- We collected a limited amount of non-invasive physiological data and did not collect data on measures such as end-tidal carbon dioxide or muscle oxygenation.
- We only included healthy young adults who exercised regularly in this study to minimise the risk of serious adverse events, but this may limit the generalisability of our findings to the wider population.

For peer review only

For peer review only - http://bmjopen.bmj.com/site/about/guidelines.xhtml

INTRODUCTION

Severe acute respiratory syndrome virus 2 (SARS-CoV-2) is an airborne virus, which causes Coronavirus Disease 2019 (COVID-19). Transmission of SARS-CoV-2 is thought to vary based on multiple factors, including host viral load, indoor versus outdoor settings, ambient air flow, volume of speech and physical proximity.[1] A number of these risk factors are relevant to exercise, with previous outbreak clusters reported in boxing or fitness gyms, where people are likely to be talking loudly or breathing heavily in a confined, indoor space.[2 3] Public health measures such as physical distancing can help reduce transmission risk,[4] but heavy breathing or panting during exercise may still increase the risk of transmitting the SARS-CoV-2 virus, even when people are exercising outdoors.[1]

Facemasks can reduce transmission of SARS-CoV-2 (Box 1). In a recent systematic review and meta-analysis, mask wearing was associated with a decrease in the incidence of COVID-19 (relative risk 0.47, 95%CI 0.29 to 0.75, I^2=84%).[5] However, there has been a lack of clear guidance on wearing facemasks during exercise and a number of recent commentary pieces have suggested that facemasks may interfere with an individual's exercise capacity.[6] Exercise leads to physiological changes, such as an increase in heart and breathing rates, to deliver more oxygen to the body. A facemask may theoretically interfere with gas exchange, potentially leading to an accumulation of carbon dioxide (CO_2) and lactate, with a reduction in oxygen saturation (SO_2).[7] This may depend in part upon the type of mask worn.[8 9] Cloth masks tend to capture moisture in exhaled breath to a greater extent than surgical or filtering face piece (FFP) respirator masks, which may particularly interfere with oxygen transfer.[10]

Box 1: Properties of different kinds of face covering

An FFP3 respirator is personal protective equipment made to an industry standard and designed for occupational health use (e.g. healthcare workers treating high-risk infectious patients). FFP3 (equivalent to the US standard N99) filters 99% of airborne particles of the relevant size.

A surgical mask is made of folded paper with waterproof backing. It is designed primarily to block egress (outward emission) of droplets such as saliva or sneezes. Surgical masks are not made to industry standards, nor are they designed to filter airborne particles. Poor

For peer review only - http://bmjopen.bmj.com/site/about/guidelines.xhtml

fit is a commonly-described problem with surgical masks, allowing air to escape through gaps.

The cloth face covering used in this study was a double-layer neck gaiter supplied by Buff©. Cloth face coverings come in many materials and thicknesses; most have not been manufactured to any filtration standard.[11] They are thought to provide partial protection of others by reducing egress of particles and droplets.[12]

The efficacy of masks depends heavily on fit and compliance.[13 14] Despite their limited efficacy, both surgical masks and cloth face coverings may contribute significantly to reducing SARS-CoV-2 transmission if a high proportion of the population wears them in high-risk settings (e.g. indoors, crowded and at times of high prevalence of Covid-19).[15 16]

In one laboratory study, a respirator reduced filtration of an artificial respiratory aerosol by 99%; a medical grade ('surgical') mask by 59% and a double neck gaiter by 60%.[17] A modelling study calculated that if two people are both wearing respirators, the risk of infection is reduced to 0.14% of the risk if neither are masked.[14]

There have been a number of recent studies assessing the impact of wearing a FFP or surgical mask during exercise, but most of these included very small numbers of participants.[7 18 19] As health and fitness facilities and physical therapy rehabilitation units re-open, it is important to determine to what extent wearing a facemask in these settings is feasible, in addition to other public health measures. We aimed to determine the safety and tolerability of healthy young adults wearing different types of facemask during moderate-to-high intensity exercise.

METHODS

Study design

We conducted a randomised crossover study, where all participants completed four x 15 minutes of moderate-to-high intensity exercise wearing three different types of facemask (cloth, surgical or FFP3) and no facemask in a random order, with a 5 minute rest period between each bout of exercise. The study team used an online simple random sequence generator to allocate mask order for each participant, without blinding.

Ethics approval

For peer review only - http://bmjopen.bmj.com/site/about/guidelines.xhtml

The University of Oxford, Central University Research Ethics Committee provided ethical approval for the study (reference R&2015/RE001).

Participants

People in the community were eligible for the study if they were aged between 18 and 35 years, exercised at least three times a week and were willing and able to give informed consent. We excluded anyone with a significant acute or longstanding medical illness that limited their exercise capacity. Volunteer participants were recruited through student groups at the University of Oxford, Nottingham University and King's College London.

When planning the study, there was only limited existing research of changes in SO_2 during exercise. A 2018 study assessed the effect of aerobic exercise on arterial blood SO_2 among 36 healthy male volunteers. Mean pre-exercise SO_2 were 97.3% (standard deviation (SD) 1.3, range 95.0% to 99.0%), compared to mean post-exercise SO_2 96.2% (2.2, 92.0% to 98.0%).[20] We made a conservative estimate that exercise with a facemask might produce a 1.5% margin of difference in peripheral SO_2. Based on a standard deviation of 2.6 and power of 80%, we calculated a minimum sample size of 55 people was needed, but aimed to recruit over 60 to allow for some participants dropping out and a 10% margin of error.

Participants could state a preference for their choice of exercise, between either running, cycling or rowing. As it turned out, all but one did running, as this allowed us to comply with restrictions on social gatherings and physical distancing that were in place because of the COVID-19 pandemic.

Outcomes

The primary outcome was change in SO_2 between different mask types. This was measured at baseline, halfway through, on completion and 1 minute after completion for each bout of exercise. Secondary outcomes included other changes in exercise performance (e.g. heart rate, distance travelled and average speed), perceived impact of mask wearing during exercise (e.g. mask comfort, ease of breathing, and ease of exercise) and willingness to wear a mask for exercise in the future.

Statistical analysis

We calculated the difference in SO_2 and heart rate (bpm) between exercising with each of the three different facemasks compared to no mask in each participant. We used differences rather than raw outcomes as these are more likely to be normally distributed. Dependency within subjects was accounted for by including a subject specific effect in a two-way Analysis of

For peer review only

Variance (ANOVA). Average differences and 95% confidence intervals for the three comparisons (cloth vs none, surgical mask vs none and FPR mask versus none) were calculated using estimated marginal means, averaging over subject. Based on prior literature, we pre-specified a non-inferiority margin of a 2% reduction in SO_2 and 7 bpm increase in heart rate. Non-inferiority was considered demonstrated if the lower bound of the 95% confidence interval did not overlap the 2% margin for SO_2 and if the upper bound of the 95% confidence interval did not overlap 7 bpm for heart rate. We conducted a sub-group analysis to check for significant changes in SO_2 or heart rate among participants with a low baseline SO_2, defined as ≤98%. Comparisons of average distance travelled and ease of breathing whilst wearing different types of facemask compared to no mask were analysed using a Bradley–Terry Model for Paired Preferences.[21] All analyses were done in R version 4.1.1 (2021-08-10)

Qualitative analysis

Free text comments were grouped into categories using a pre-defined framework, based around positive, negative and neutral comments for each type of mask. Using a comment-by-comment coding approach in Excel, we then undertook thematic analysis to identify and group common threads within these categories, seeking emergent as well as anticipated themes.[22 23]

Patient and public involvement

No patients involved.

RESULTS

In total, 72 participants completed the study between June 2021 and January 2022, including 33 women (46%) and 39 men (54%). The mean age was 23.9 years (SD 3.55). Most participants exercised 3-4 (n=38, 54%) or 5-6 times per week (n=30, 42%), but 3 participants (4%) exercised every day. One participant was a smoker.

All 72 participants completed all four exercise sessions. One participant completed the exercise on an indoor rowing machine and 71 undertook running outdoors.

Oxygen saturations during exercise

Changes in SO_2 did not exceed the non-inferiority margin at any point of exercise wearing any of the three masks (Table 1). There was a small but statistically significant decrease in

For peer review only - http://bmjopen.bmj.com/site/about/guidelines.xhtml

SO_2 during exercise wearing either a cloth mask (estimated average difference (EAD) -0.78%, 95%CI -1.12 to -0.44) or the FFP3 compared to no mask (-0.82%, -1.16 to -0.47), but not when wearing the surgical mask (-0.28%, -0.63 to 0.07). There was no difference in SO_2 comparing any mask type to no mask after completing exercise.

Table 1. Changes in oxygen saturations comparing different types of facemask worn during exercise

Contrast	Average oxygen saturations (%) with mask	Average oxygen saturations (%), no mask	Estimated average difference	Lower 95 confidence limit	Upper 95 confidence limit	Non-inferior @ -2%
During exercise (N = 80)						
Cloth - None	96.5	97.4	-0.90	-1.48	-0.32	TRUE
Surgical - None	97.3	97.4	-0.12	-0.72	0.49	TRUE
*FFP - None	96.5	97.4	-0.88	-1.47	-0.29	TRUE
End of exercise (N = 78)						
Cloth - None	96.9	97.0	-0.07	-0.57	0.43	TRUE
Surgical - None	97.0	97.0	-0.03	-0.55	0.49	TRUE
*FFP - None	97.0	97.0	0.00	-0.52	0.52	TRUE
1 minute after end of exercise (N = 74)						
Cloth - None	97.6	97.7	-0.11	-0.61	0.39	TRUE
Surgical - None	97.9	97.7	0.18	-0.32	0.67	TRUE
*FFP - None	97.6	97.7	-0.08	-0.57	0.42	TRUE

*FFP = filtering face piece respirator masks

In a sub-group analysis among those with lower baseline $SO_{2,}$ changes in SO_2 still did not exceed the non-inferiority margin of 2% although SO_2 during exercise was 1% lower among people wearing either the FFP3 (EAD -0.88, 95%CI -1.47 to -0.29) or cloth mask (-0.90, -1.48 to -0.32) (Supplemental table 1).

For peer review only - http://bmjopen.bmj.com/site/about/guidelines.xhtml

Changes in heart rate, performance or additional symptoms during exercise

There was no difference in heart rate at any time point comparing the three types of mask to no mask (Table 2) and changes in heart rate did not exceed the non-inferiority margin.

Table 2. Changes in heart rate comparing different types of facemask worn during exercise

Contrast	Average heart rate (BPM), with mask	Average heart rate (BPM), no mask	Estimated average difference	Lower 95 confidence limit	Upper 95 confidence limit	Non-inferior @ +7bpm
During exercise (N = 69)						
Cloth - None	157.5	157.3	0.10	-2.90	3.10	TRUE
Surgical - None	157.8	157.3	0.78	-2.33	3.89	TRUE
FFP - None	159.9	157.3	2.84	-0.24	5.91	TRUE
End of exercise (N = 69)						
Cloth - None	156.9	158.2	-1.20	-4.56	2.15	TRUE
Surgical - None	158.7	158.2	0.36	-3.01	3.73	TRUE
FFP - None	158.9	158.2	0.52	-2.85	3.89	TRUE
1 minute after the end of exercise (N = 67)						
Cloth - None	124.8	124.2	0.40	-2.15	2.95	TRUE
Surgical - None	124.6	124.2	0.49	-2.03	3.02	TRUE
FFP - None	125.3	124.2	1.45	-1.11	4.01	TRUE

*BPM = beats per minute. FFP = filtering face piece respirator masks

Among the subgroup with $SO_2 \leq 98\%$ at baseline, there was no significant difference in average heart rate during or after exercise with any facemask (Supplemental table 2). However, the upper 95% CI did exceed the non-inferiority margin of 7bpm for the FFP3 mask compared to no mask at each time point measured. This was also true for the surgical mask at the end of exercise and after a 1 minute rest period.

Across participants there was no significant difference in average speed or distance travelled when comparing any of the facemasks to no mask (Table 3).

Table 3. Comparison of average distance travelled whilst wearing different types of facemask compared to no mask

Contrast	Average distance travelled (km) with mask	Average distance travelled (km), no mask	Estimated average difference	Lower 95 confidence limit	Upper 95 confidence limit
Distance travelled (km) during exercise (N = 71)					
Cloth mask - No facemask	2.75	2.82	-0.07	-0.15	0.01
Surgical mask - No facemask	2.78	2.82	-0.04	-0.12	0.04
FFP mask - No facemask	2.76	2.82	-0.06	-0.14	0.02

* FFP = filtering face piece respirator masks

The most common symptoms participants reported feeling wearing a facemask during exercise were increased breathlessness (n=13, 18%), dizziness (n=7, 10%), ‘other’ symptoms (n=7, 10%) and fatigue (n=6, 8%). Four participants found wearing a mask claustrophobic and one developed a headache. No participants reported feeling anxious or drowsy and none had to stop during exercise due to side-effects.

Perception of comfort, breathing and ease of exercise

When asked which facemask they found most difficult to exercise wearing, 40 (56%) participants said the cloth mask, 27 (38%) said FFP3 and 4 (6%) said the surgical mask. Participants felt that the surgical mask was the most comfortable (Figure 1) and the easiest to breathe through (Figure 2). In a direct comparison, the probability of a surgical mask being ranked easier to exercise in than a cloth mask was 0.81 (95%CI 0.72 to 0.88, p=0.00) and being easier than FFP3 was 0.80 (0.73 to 0.86, p=0.00) (Table 4). Surgical masks were also ranked easier to breathe through than the cloth mask (probability 0.86, 95%CI 0.78 to 0.91) or FFP3 (0.83, 0.76 to 0.88). The FFP3 and cloth mask were ranked equal for ease of exercise and breathing through.

Table 4. Comparison of reported comfort, ease of breathing and ease of overall exercise between the different types of facemask worn during exercise

For peer review only - http://bmjopen.bmj.com/site/about/guidelines.xhtml

Type 1	Type 2	Number rating Type 1 lower	Number rating Type 2 lower	Probability type 1 rated lower	P value	Lower 95 confidence limit	Upper 95 confidence limit
Ease of Exercise, lower score = easier							
FFP	Surgical[1]	12	51	0.20	0.00	0.13	0.29
Surgical	Cloth	50	12	0.81	0.00	0.72	0.88
Cloth	FFP[2]	29	31	0.49	0.84	0.38	0.60
Ease of Breathing, lower score = easier							
FFP	Surgical[1]	11	55	0.17	0.00	0.11	0.26
Surgical	Cloth	59	10	0.86	0.00	0.78	0.91
Cloth	FFP[2]	28	36	0.44	0.30	0.34	0.55

[1]Surgical is ranked 1st

[2]FFP and cloth are equal 2nd

*FFP = filtering face piece respirator masks

In free text comments, participants commented on the cloth mask becoming *'soaked through with sweat and water vapour'*. A number commented on how the cloth mask '*sucks in when breathing in'* or '*kept getting in my mouth'*, whilst others found it '*kept slipping down'*. One participant described running in the cloth mask as *'like being waterboarded'*. There were 35 negative free text comments about the cloth mask and one positive comment.

There were 15 negative free text comments about the FFP3 mask, which some participants found '*constraining*', '*so tight fitting it felt difficult to breathe'* and caused '*nose pain'*. Some people found this made them feel '*a bit dizzy'*, '*really lightheaded'*, or '*as though my heart was going really high when I was wearing it*'. However, there were also 10 positive comments about wearing the FFP3, with these participants reporting '*a good fit'*, which '*was very comfortable'*, '*amenable to glasses-wearing'* and which '*didn't shake at all whilst running, unlike the others*'.

There were 11 positive comments about wearing the surgical mask, mostly reporting it was '*fine*', the *'easiest one'* or caused '*little restriction'*. The four negative comments about the surgical mask focused on glasses steaming up or the mask slipping.

Willingness to wear a facemask for exercise in the future

There were 33 participants who were accepting of the idea of mandatory facemasks, 22 who were opposed and 17 who gave more neutral responses. Positive replies included '*It would*

make no real impact to my exercise having to wear a facemask', '*Wouldn't bother me too much'* and '*To be honest, if wearing a facemask during exercise will limit the spread of COVID then I am happy to do so'*. A number of participants mentioned they '*wouldn't mind doing so indoors'*, or '*only if it was the surgical mask'*. In the neutral group there were comments such as '*I would tolerate it but not advocate it'* and '*I would if I had to'*. Among those opposed people mentioned '*I would be annoyed, because I don't think that it's really needed for preventing transmission, especially outdoors'* and that they '*would rather have a choice in the matter'*. Some people compared favourably the experience of exercising with no mask compared to wearing any mask, with one saying '*When I ran with no mask at the end it felt like breathing in HD'*.

DISCUSSION

We found no clinically significant reduction in SO_2 or heart rate with any of the three facemasks, suggesting that exercising at moderate-to-high intensity wearing a facemask appears to be safe in healthy, young adults. However, around one in three participants reported additional symptoms when wearing a facemask, including feeling more breathless, dizzy or fatigued. Surgical facemasks were experienced as the most comfortable, easiest to breathe through and had the least impact on exercise, though importantly we did not measure whether these masks were well-fitting and poor fit of surgical masks is common and greatly reduces protection.[14] There was most support for wearing a surgical facemask during indoor exercise if needed to reduce the spread of COVID-19, though around a third of participants were opposed to mandatory wearing of facemasks during exercise.

Strengths and limitations

To our knowledge, this is the largest study to date to investigate the safety and tolerability of wearing a facemask during exercise. We used a randomised crossover design to ensure that the effect of any fatigue from exercise was equal between mask types. This also allowed each participant to complete the study in a single session, minimising the risk of recall bias when completing the study questionnaire. We asked participants to exercise at a consistent speed between study sessions and recorded their average speed and distance travelled, with no significant difference in exercise performance based on type of facemask worn.

For peer review only - http://bmjopen.bmj.com/site/about/guidelines.xhtml

We chose to recruit only healthy young participants to reduce the risk of serious adverse events, because of the limited pre-existing data demonstrating wearing a facemask during exercise was safe. However, our results may not be generalisable to people who have underlying health conditions or restricted exercise capacity and further research is needed in these populations.

We collected a limited amount of non-invasive physiological data from participants. Healthy young participants may maintain a constant SO_2 and heart rate through compensatory mechanisms, such as increased respiratory rate, which we have not captured in our analysis. However, all of our participants were able to complete the exercise wearing each mask type with no significant change in their speed or distance travelled, supporting the finding that healthy young people can safely exercise wearing facemasks.

Because of the COVID-19 pandemic and restrictions in social interaction, we conducted almost all of the study sessions outdoors. During cold weather some participants had cold peripheries whilst exercising, meaning the pulse oximeters were not always able to record SO_2 and heart rate or were slow to do so. Delays in obtaining accurate oximeter readings post-exercise may have led to some recovery in heart rate and SO_2, which would result in an under-estimate of any change in these parameters.

Comparison with other studies

Our results support the findings of a recent systematic review and meta-analysis, which assessed the impact of wearing a facemask during exercise.[24] This reported no significant difference in exercise performance among studies of surgical facemasks (standard mean difference (SMD) -0.05, 95CI -0.16 to 0.07) or N95 masks (-0.16, -0.54 to 0.22).[24] Patients did have increased ratings of perceived exertion (0.33, 0.09 to 0.58) and dyspnoea (0.60, 0.30 to 0.90) with all masks.[24] In contrast to this review, we include cloth masks in the comparison of facemasks types. The exercise tested in these studies was heterogeneous, ranging from 1 hour treadmill walks at low intensity, to strength training with half-squats and progressive exercise training on a bicycle. Our results demonstrate the principal findings of this review are applicable to healthy young adults during moderate-to-high intensity exercise.

A second recent systematic review of facemask wearing during rest, work or exercise identified 14 crossover trials with 25 independent intervention arms.[25] There were a total of 246 participants across these studies. High intensity exercise was associated with a small

For peer review only - http://bmjopen.bmj.com/site/about/guidelines.xhtml

reduction in SO_2 (SMD -0.40, 95%CI -0.70 to – 0.09) and lower respiratory rates (-0.25, -0.44 to -0.06), particularly when wearing a FFP2 or N95 facemask.[25] However, there was no association with mask wearing and change in exercise performance, heart rate or exhaled CO_2. This review suggests that undertaking exhausting exercise wearing FFP-type facemasks may reduce SO_2 but supports our finding that mask wearing appears to be safe and does not negatively affect overall performance.

Implications for policy and research

Governments globally are having to decide which public health measures to continue in response to changing incidence of COVID-19. Interventions that can contribute towards reducing transmission, whilst allowing people to return to normal life, are particularly important. Facemasks can reduce transmission of the SARS-CoV-2 virus and appear to be safe to wear among young, healthy adults during exercise. Public health messaging could support their use in higher risk settings, such as indoor settings with poor ventilation or outdoor exercise among large volumes of people. Surgical masks appear to be the least restrictive type of mask to wear and may help reduce transmission by preventing the forward propulsion of a large jet of exhaled aerosol particles. However, the comparative effectiveness of different mask types in reducing the risk of transmission of SARS-CoV-2 remains unknown and could be a focus for future research.

Wearing a facemask during exercise does not negatively impact exercise performance. It is possible there may even be benefits for longer-term endurance exercise performance if facemask wearing did induce mild hypoxia. Hypoxic exercise training has previously been reported to improve cardiac haemodynamics and exercise capacity,[26 27] though these studies typically recruited only small numbers of participants and have not been consistently replicated.[28] Further research into the long-term impact on exercise performance of wearing a facemask during exercise could help better inform athletes of the relative risks and benefits of this intervention.

CONCLUSION

In this randomised crossover trial we found that healthy young adults were able to exercise at moderate-to-high intensity wearing either a surgical, cloth or FFP facemask without significant restriction on exercise performance or physiological limitation. However, wearing

For peer review only - http://bmjopen.bmj.com/site/about/guidelines.xhtml

a facemask during exercise made some participants feel more breathless, dizzy or fatigued and most people felt it was less comfortable and enjoyable than exercising without a mask. There was limited support for wearing a facemask to exercise across all settings, but these results could support the use of facemask wearing whilst exercising in a limited number of high-risk settings as a safe intervention to reduce the risk of SARS-CoV-2 transmission.

For peer review only

Registration

The trail was registered in advance on ClinicalTrials.gov on 21st June 2021, 'Face Masks and Oxygen Levels – What Happens During Exercise? (Masks in Exercise – a Randomised Trial) (MERIT). The NCT number is NCT04932226.

Contributors

NRJ and TG conceived the study. NRJ, TG and HPD wrote the protocol. NRJ, SM, KN and JB conducted all study visits. JO and NRJ carried out the analysis. NRJ wrote the first draft of the manuscript and is the guarantor and corresponding author. All authors contributed to the interpretation of the results and revisions of the manuscript. The corresponding author attests that all listed authors meet authorship criteria and that no others meeting the criteria have been omitted.

Acknowledgements

Thanks to Robert Temple who also helped with data collection on one study visit.

Competing interests

No competing interests.

Funding

Buff© provided 60 original tubular cotton neckwear free of charge, which were used as the cloth masks. Buff© had no role in study design, data collection, data analysis, data interpretation, writing of the report, or any other aspect of the study.

NRJ is supported by a Wellcome Trust Doctoral Research Fellowship (203921/Z/16/Z). FDRH acknowledges his part-funding from the NIHR Applied Research Collaboration (ARC) Oxford Thames Valley, the NIHR Oxford Biomedical Research Centre (BRC), and

For peer review only - http://bmjopen.bmj.com/site/about/guidelines.xhtml

the NIHR Oxford Medtech and In-Vitro Diagnostics Co-operative. TG's research is supported by Oxford NIHR Biomedical Research Centre (grant no BRC-1215-20008).

Data sharing

To request access to deidentified participant data or the study protocol, please contact the lead author.

For peer review only

REFRENCES

1. Jones NR, Qureshi ZU, Temple RJ, et al. Two metres or one: what is the evidence for physical distancing in covid-19? *BMJ* 2020;370:m3223. doi: 10.1136/bmj.m3223 [published Online First: 2020/08/28]
2. Chu DKW, Gu H, Chang LDJ, et al. SARS-CoV-2 Superspread in Fitness Center, Hong Kong, China, March 2021. *Emerg Infect Dis* 2021;27(8):2230-32. doi: 10.3201/eid2708.210833 [published Online First: 2021/05/19]
3. Jang S, Han SH, Rhee JY. Cluster of Coronavirus Disease Associated with Fitness Dance Classes, South Korea. *Emerg Infect Dis* 2020;26(8):1917-20. doi: 10.3201/eid2608.200633 [published Online First: 2020/05/16]
4. Helsingen LM, Loberg M, Refsum E, et al. Covid-19 transmission in fitness centers in Norway - a randomized trial. *BMC Public Health* 2021;21(1):2103. doi: 10.1186/s12889-021-12073-0 [published Online First: 2021/11/19]
5. Talic S, Shah S, Wild H, et al. Effectiveness of public health measures in reducing the incidence of covid-19, SARS-CoV-2 transmission, and covid-19 mortality: systematic review and meta-analysis. *BMJ* 2021;375:e068302. doi: 10.1136/bmj-2021-068302 [published Online First: 2021/11/19]
6. Bottoms L. Why it could be dangerous to exercise with a face mask on: The Conversation; 2020 [Available from: https://theconversation.com/why-it-could-be-dangerous-to-exercise-with-a-face-mask-on-140277 accessed 05.08.2020.
7. Fikenzer S, Uhe T, Lavall D, et al. Effects of surgical and FFP2/N95 face masks on cardiopulmonary exercise capacity. *Clin Res Cardiol* 2020 doi: 10.1007/s00392-020-01704-y [published Online First: 2020/07/08]
8. Li Y, Tokura H, Guo YP, et al. Effects of wearing N95 and surgical facemasks on heart rate, thermal stress and subjective sensations. *Int Arch Occup Environ Health* 2005;78(6):501-9. doi: 10.1007/s00420-004-0584-4 [published Online First: 2005/05/27]
9. Person E, Lemercier C, Royer A, et al. [Effect of a surgical mask on six minute walking distance]. *Rev Mal Respir* 2018;35(3):264-68. doi: 10.1016/j.rmr.2017.01.010 [published Online First: 2018/02/06]
10. MacIntyre CR, Seale H, Dung TC, et al. A cluster randomised trial of cloth masks compared with medical masks in healthcare workers. *BMJ Open* 2015;5(4):e006577. doi: 10.1136/bmjopen-2014-006577 [published Online First: 2015/04/24]
11. Konda A, Prakash A, Moss GA, et al. Aerosol Filtration Efficiency of Common Fabrics Used in Respiratory Cloth Masks. *ACS Nano* 2020;14(5):6339-47. doi: 10.1021/acsnano.0c03252 [published Online First: 2020/04/25]
12. Clase CM, Fu EL, Ashur A, et al. Forgotten Technology in the COVID-19 Pandemic: Filtration Properties of Cloth and Cloth Masks-A Narrative Review. *Mayo Clin Proc* 2020;95(10):2204-24. doi: 10.1016/j.mayocp.2020.07.020 [published Online First: 2020/10/06]
13. Czypionka T, Greenhalgh T, Bassler D, et al. Masks and Face Coverings for the Lay Public : A Narrative Update. *Ann Intern Med* 2021;174(4):511-20. doi: 10.7326/M20-6625 [published Online First: 2020/12/29]
14. Bagheri G, Thiede B, Hejazi B, et al. An upper bound on one-to-one exposure to infectious human respiratory particles. *Proc Natl Acad Sci U S A* 2021;118(49) doi: 10.1073/pnas.2110117118 [published Online First: 2021/12/04]
15. Cheng Y, Ma N, Witt C, et al. Face masks effectively limit the probability of SARS-CoV-2 transmission. *Science* 2021 doi: 10.1126/science.abg6296 [published Online First: 2021/05/22]
16. Ku D, Yeon C, Lee S, et al. Safe traveling in public transport amid COVID-19. *Sci Adv* 2021;7(43):eabg3691. doi: 10.1126/sciadv.abg3691 [published Online First: 2021/10/23]

For peer review only - http://bmjopen.bmj.com/site/about/guidelines.xhtml

17. Lindsley WG, Beezhold DH, Coyle J, et al. Efficacy of universal masking for source control and personal protection from simulated cough and exhaled aerosols in a room. *J Occup Environ Hyg* 2021;18(8):409-22. doi: 10.1080/15459624.2021.1939879 [published Online First: 2021/06/24]
18. Mapelli M, Salvioni E, De Martino F, et al. "You can leave your mask on": effects on cardiopulmonary parameters of different airway protective masks at rest and during maximal exercise. *Eur Respir J* 2021;58(3) doi: 10.1183/13993003.04473-2020 [published Online First: 2021/03/09]
19. Kim JH, Benson SM, Roberge RJ. Pulmonary and heart rate responses to wearing N95 filtering facepiece respirators. *Am J Infect Control* 2013;41(1):24-7. doi: 10.1016/j.ajic.2012.02.037 [published Online First: 2012/09/05]
20. Eroğlu H, Okyaz B, Türkçapar U. The Effect of Acute Aerobical Exercise on Arterial Blood Oxygen Saturation of Athletes. *Journal of Education and Training Studies* 2018;6:74-79. doi: 10.11114/jets.v6i9a.3562
21. Turner H, Firth D. Bradley-Terry Models in R: The BradleyTerry2 Package. *Journal of Statistical Software* 2012;48(9):1–21. doi: 10.18637/jss.v048.i09
22. Braun V, Clarke V. Using thematic analysis in psychology. *Qualitative Research in Psychology* 2006;3(2):77-101. doi: 10.1191/1478088706qp063oa
23. Ziebland S, McPherson A. Making sense of qualitative data analysis: an introduction with illustrations from DIPEx (personal experiences of health and illness). *Med Educ* 2006;40(5):405-14. doi: 10.1111/j.1365-2929.2006.02467.x [published Online First: 2006/04/26]
24. Shaw KA, Zello GA, Butcher SJ, et al. The impact of face masks on performance and physiological outcomes during exercise: a systematic review and meta-analysis. *Appl Physiol Nutr Metab* 2021;46(7):693-703. doi: 10.1139/apnm-2021-0143 [published Online First: 2021/04/27]
25. Engeroff T, Groneberg DA, Niederer D. The Impact of Ubiquitous Face Masks and Filtering Face Piece Application During Rest, Work and Exercise on Gas Exchange, Pulmonary Function and Physical Performance: A Systematic Review with Meta-analysis. *Sports Med Open* 2021;7(1):92. doi: 10.1186/s40798-021-00388-6 [published Online First: 2021/12/14]
26. Chacaroun S, Borowik A, Vega-Escamilla YGI, et al. Hypoxic Exercise Training to Improve Exercise Capacity in Obese Individuals. *Med Sci Sports Exerc* 2020;52(8):1641-49. doi: 10.1249/MSS.0000000000002322 [published Online First: 2020/02/27]
27. Wang JS, Lee MY, Lien HY, et al. Hypoxic exercise training improves cardiac/muscular hemodynamics and is associated with modulated circulating progenitor cells in sedentary men. *Int J Cardiol* 2014;170(3):315-23. doi: 10.1016/j.ijcard.2013.11.005 [published Online First: 2013/11/30]
28. Vogt M, Hoppeler H. Is hypoxia training good for muscles and exercise performance? *Prog Cardiovasc Dis* 2010;52(6):525-33. doi: 10.1016/j.pcad.2010.02.013 [published Online First: 2010/04/27]

For peer review only - http://bmjopen.bmj.com/site/about/guidelines.xhtml

Figure legends

Figure 1. Reported comfort of wearing different types of facemask during exercise

Caption: The graph plots the number of participants who reported each comfort score, between 1 and 9, for each of the three different types of facemask.

Figure 2. Reported ease of breathing wearing different types of facemask during exercise

Caption: The graph plots the number of participants who reported each ease of breathing score, between 1 and 9, for each of the three different types of facemask.

For peer review only

For peer review only - http://bmjopen.bmj.com/site/about/guidelines.xhtml

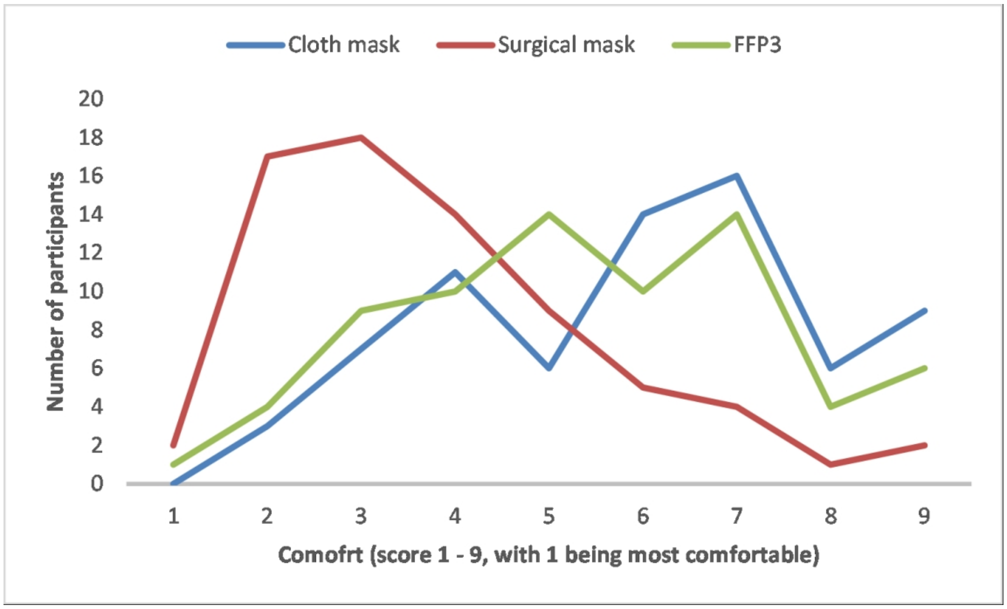

Figure 1. Reported comfort of wearing different types of facemask during exercise
Caption: The graph plots the number of participants who reported each comfort score, between 1 and 9, for each of the three different types of facemask.

127x76mm (300 x 300 DPI)

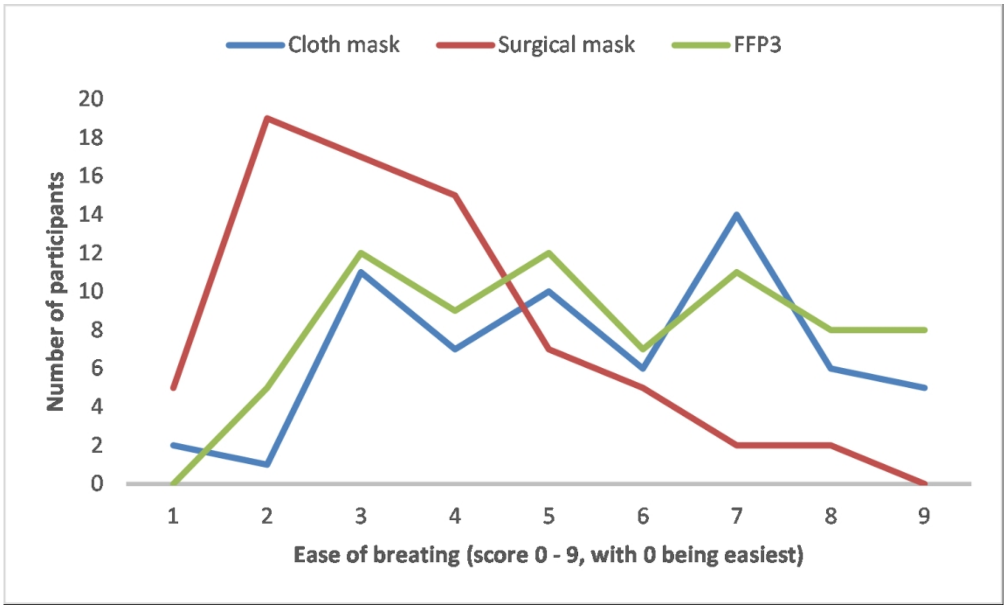

Figure 2. Reported ease of breathing wearing different types of facemask during exercise
Caption: The graph plots the number of participants who reported each ease of breathing score, between 1 and 9, for each of the three different types of facemask.

127x76mm (300 x 300 DPI)

Supplemental Table 1a. Changes in oxygen saturations comparing different mask types worn during exercise. Sub-group analysis of participants with resting oxygen saturations of ≤ 98%

Contrast	Average oxygen saturations (%) with mask	Average oxygen saturations (%), no mask	Estimated average difference	Lower 95 confidence limit	Upper 95 confidence limit	Non-inferior @ -2%
During exercise (N = 30)						
Cloth - None	96.5	97.4	-0.90	-1.48	-0.32	TRUE
Surgical - None	97.3	97.4	-0.12	-0.72	0.49	TRUE
FFP - None	96.5	97.4	-0.88	-1.47	-0.29	TRUE
End of exercise (N = 29)						
Cloth - None	96.9	97.0	-0.07	-0.57	0.43	TRUE
Surgical - None	97.0	97.0	-0.03	-0.55	0.49	TRUE
FFP - None	97.0	97.0	0.00	-0.52	0.52	TRUE
1 minute after the end of exercise (N = 30)						
Cloth - None	97.6	97.7	-0.11	-0.61	0.39	TRUE
Surgical - None	97.9	97.7	0.18	-0.32	0.67	TRUE
FFP - None	97.6	97.7	-0.08	-0.57	0.42	TRUE

*FFP = filtering face piece respirator masks

Supplemental Table 1b. Changes in oxygen saturations comparing different mask types worn during exercise. Sub-group analysis of participants with resting oxygen saturations of > 98%

Contrast	Average oxygen saturations (%) with mask	Average oxygen saturations (%), no mask	Estimated average difference	Lower 95 confidence limit	Upper 95 confidence limit	Non-inferior @ -2%
During exercise (N = 39)						
Cloth - None	97.3	97.9	-0.69	-1.11	-0.27	TRUE
Surgical - None	97.5	97.9	-0.40	-0.83	0.03	TRUE
FFP - None	97.2	97.9	-0.77	-1.19	-0.35	TRUE
End of exercise (N = 39)						
Cloth - None	97.2	97.2	-0.07	-0.48	0.35	TRUE
Surgical - None	97.7	97.2	0.51	0.11	0.92	TRUE
FFP - None	96.9	97.2	-0.36	-0.76	0.05	TRUE
1 minute after the end of exercise (N = 37)						
Cloth - None	97.8	97.7	0.16	-0.18	0.49	TRUE
Surgical - None	98.2	97.7	0.46	0.13	0.79	TRUE
FFP - None	98.1	97.7	0.32	-0.01	0.66	TRUE

*****FFP = filtering face piece respirator masks

Supplemental Table 2a. Changes in heart rate comparing different mask types worn during exercise. Sub-group analysis of participants with resting oxygen saturations of ≤ 98%

Contrast	Average heart rate (%) with mask	Average heart rate (%), no mask	Estimated average difference	Lower 95 confidence limit	Upper 95 confidence limit	Non-inferior @ +7bpm
During exercise (N = 29)						
Cloth - None	156.4	158.0	-1.69	-6.55	3.17	TRUE
Surgical - None	155.9	158.0	-1.41	-6.57	3.75	TRUE
FFP - None	159.5	158.0	2.21	-2.95	7.37	FALSE
End of exercise (N = 29)						
Cloth - None	151.5	155.9	-4.17	-10.89	2.54	TRUE
Surgical - None	156.5	155.9	0.36	-6.59	7.31	FALSE
FFP - None	158.4	155.9	2.04	-4.91	8.99	FALSE
1 minute after the end of exercise (N = 29)						
Cloth - None	123.5	123.2	0.15	-4.47	4.77	TRUE
Surgical - None	125.4	123.2	2.74	-1.88	7.36	FALSE
FFP - None	125.7	123.2	2.82	-1.96	7.60	FALSE

***** BPM = beats per minute; FFP = filtering face piece respirator masks

Supplemental Table 2b. Changes in heart rate comparing different mask types worn during exercise. Sub-group analysis of participants with resting oxygen saturations of > 98%

Contrast	Average heart rate (%) with mask (bpm)	Average heart rate (%), no mask (bpm)	Estimated average difference	Lower 95 confidence limit	Upper 95 confidence limit	Non-inferior @ +7bpm
During exercise (N = 39)						
Cloth - None	158.4	156.8	1.59	-2.22	5.40	TRUE
Surgical - None	160.1	156.8	3.39	-0.49	7.27	FALSE
FFP - None	161.6	156.8	4.74	0.93	8.55	FALSE
End of exercise (N = 39)						
Cloth - None	162.2	159.9	2.11	-1.03	5.26	TRUE
Surgical - None	160.5	159.9	0.54	-2.54	3.62	TRUE
FFP - None	159.8	159.9	-0.18	-3.26	2.90	TRUE
1 minute after the end of exercise (N = 37)						
Cloth - None	126.2	125.3	0.71	-2.31	3.73	TRUE
Surgical - None	124.5	125.3	-0.97	-3.93	1.99	TRUE
FFP - None	125.7	125.3	0.73	-2.23	3.69	TRUE

*****BPM = beats per minute; FFP = filtering face piece respirator masks

CONSORT 2010 checklist of information to include when reporting a randomised trial*

Section/Topic	Item No	Checklist item	Reported on page No
**Title and abstract**			
	1a	Identification as a randomised trial in the title	1
	1b	Structured summary of trial design, methods, results, and conclusions (for specific guidance see CONSORT for abstracts)	2
**Introduction**			
Background and objectives	2a	Scientific background and explanation of rationale	4
	2b	Specific objectives or hypotheses	5
**Methods**			
Trial design	3a	Description of trial design (such as parallel, factorial) including allocation ratio	5
	3b	Important changes to methods after trial commencement (such as eligibility criteria), with reasons	6
Participants	4a	Eligibility criteria for participants	6
	4b	Settings and locations where the data were collected	6
Interventions	5	The interventions for each group with sufficient details to allow replication, including how and when they were actually administered	6
Outcomes	6a	Completely defined pre-specified primary and secondary outcome measures, including how and when they were assessed	6
	6b	Any changes to trial outcomes after the trial commenced, with reasons	N/A
Sample size	7a	How sample size was determined	6
	7b	When applicable, explanation of any interim analyses and stopping guidelines	N/A
Randomisation:			
Sequence generation	8a	Method used to generate the random allocation sequence	5
	8b	Type of randomisation; details of any restriction (such as blocking and block size)	5
Allocation concealment mechanism	9	Mechanism used to implement the random allocation sequence (such as sequentially numbered containers), describing any steps taken to conceal the sequence until interventions were assigned	5
Implementation	10	Who generated the random allocation sequence, who enrolled participants, and who assigned participants to interventions	5
	11a	If done, who was blinded after assignment to interventions (for example, participants, care providers, those	N/A

For peer review only

For peer review only - http://bmjopen.bmj.com/site/about/guidelines.xhtml

		assessing outcomes) and how	
	11b	If relevant, description of the similarity of interventions	6
	12a	Statistical methods used to compare groups for primary and secondary outcomes	6-7
	12b	Methods for additional analyses, such as subgroup analyses and adjusted analyses	7
	13a	For each group, the numbers of participants who were randomly assigned, received intended treatment, and were analysed for the primary outcome	7
	13b	For each group, losses and exclusions after randomisation, together with reasons	All volunteers enrolled
Recruitment	14a	Dates defining the periods of recruitment and follow-up	7
	14b	Why the trial ended or was stopped	Met sample size
Baseline data	15	A table showing baseline demographic and clinical characteristics for each group	Information provided in text, page 7
Numbers analysed	16	For each group, number of participants (denominator) included in each analysis and whether the analysis was by original assigned groups	Table 1, 2
Outcomes and estimation	17a	For each primary and secondary outcome, results for each group, and the estimated effect size and its precision (such as 95% confidence interval)	Table 1-4
	17b	For binary outcomes, presentation of both absolute and relative effect sizes is recommended	N/A
Ancillary analyses	18	Results of any other analyses performed, including subgroup analyses and adjusted analyses, distinguishing pre-specified from exploratory	Supplemental tables 1 & 2
Harms	19	All important harms or unintended effects in each group (for specific guidance see CONSORT for harms)	10
**Discussion**			
Limitations	20	Trial limitations, addressing sources of potential bias, imprecision, and, if relevant, multiplicity of analyses	12-13
Generalisability	21	Generalisability (external validity, applicability) of the trial findings	13-14
Interpretation	22	Interpretation consistent with results, balancing benefits and harms, and considering other relevant evidence	13-14
**Other information**			
Registration	23	Registration number and name of trial registry	16
Protocol	24	Where the full trial protocol can be accessed, if available	N/A
Funding	25	Sources of funding and other support (such as supply of drugs), role of funders	16-17

For peer review only - http://bmjopen.bmj.com/site/about/guidelines.xhtml
For peer review only

*We strongly recommend reading this statement in conjunction with the CONSORT 2010 Explanation and Elaboration for important clarifications on all the items. If relevant, we also recommend reading CONSORT extensions for cluster randomised trials, non-inferiority and equivalence trials, non-pharmacological treatments, herbal interventions, and pragmatic trials. Additional extensions are forthcoming: for those and for up to date references relevant to this checklist, see www.consort-statement.org.

For peer review only

For peer review only - http://bmjopen.bmj.com/site/about/guidelines.xhtml

BMJ Open

FaceMasks whilst ExeRcIsing Trial (MERIT): a crossover randomised controlled study

Journal:	*BMJ Open*
Manuscript ID	bmjopen-2022-063014.R1
Article Type:	Original research
Date Submitted by the Author:	26-Sep-2022
Complete List of Authors:	Jones, Nicholas; University of Oxford, Primary Care Health Sciences<br>Oke, Jason; University of Oxford, Nuffiled Department of Primary Care Health Sciences<br>Marsh, Seren; University of Oxford<br>Nikbin, Kurosh; Kings College London<br>Bowley, Jonathan; University of Nottingham<br>Dijkstra, H Paul ; University of Oxford, Department of Continuing Education ; Aspetar Orthopaedic and Sports Medicine Hospital, Medical Education Department<br>Hobbs, Richard; University of Oxford, Dept of Primary Health Care<br>Greenhalgh, Trisha; University of Oxford, Nuffield Department of Primary Care Health Sciences
**Primary Subject Heading**:	Public health
Secondary Subject Heading:	Sports and exercise medicine, Infectious diseases
Keywords:	COVID-19, Public health < INFECTIOUS DISEASES, PREVENTIVE MEDICINE

SCHOLARONE™
Manuscripts

I, the Submitting Author has the right to grant and does grant on behalf of all authors of the Work (as defined in the below author licence), an exclusive licence and/or a non-exclusive licence for contributions from authors who are: i) UK Crown employees; ii) where BMJ has agreed a CC-BY licence shall apply, and/or iii) in accordance with the terms applicable for US Federal Government officers or employees acting as part of their official duties; on a worldwide, perpetual, irrevocable, royalty-free basis to BMJ Publishing Group Ltd ("BMJ") its licensees and where the relevant Journal is co-owned by BMJ to the co-owners of the Journal, to publish the Work in this journal and any other BMJ products and to exploit all rights, as set out in our licence.

The Submitting Author accepts and understands that any supply made under these terms is made by BMJ to the Submitting Author unless you are acting as an employee on behalf of your employer or a postgraduate student of an affiliated institution which is paying any applicable article publishing charge ("APC") for Open Access articles. Where the Submitting Author wishes to make the Work available on an Open Access basis (and intends to pay the relevant APC), the terms of reuse of such Open Access shall be governed by a Creative Commons licence – details of these licences and which Creative Commons licence will apply to this Work are set out in our licence referred to above.

Other than as permitted in any relevant BMJ Author's Self Archiving Policies, I confirm this Work has not been accepted for publication elsewhere, is not being considered for publication elsewhere and does not duplicate material already published. I confirm all authors consent to publication of this Work and authorise the granting of this licence.

For peer review only

FaceMasks whilst ExeRcIsing Trial (MERIT): a crossover randomised controlled study

[1]Nicholas R Jones
[1]Jason L. Oke
[2]Seren Marsh
[3]Kurosh Nikbin
[4]Jonathan Bowley
[5,6]H Paul Dijkstra
[1]FD Richard Hobbs
[1]Trisha Greenhalgh

1. Nuffield Department of Primary Care Health Sciences, University of Oxford, Oxford, UK
2. University of Oxford Medical School, University of Oxford, Oxford, UK
3. GKT School of Medical Education, Kings College London, London, UK
4. School of Medicine, University of Nottingham, Nottingham, UK
5. Medical Education Department, Aspetar Orthopaedic and Sports Medicine Hospital, Doha, Qatar
6. Department for Continuing Education, University of Oxford, Oxford, UK

Corresponding author: Dr Nicholas Jones, Nuffield Department of Primary Care Health Sciences, University of Oxford, Radcliffe Primary Care Building, Woodstock Road, Oxford OX2 6GG.

Email: nicholas.jones2@phc.ox.ac.uk. Twitter @drnrjones. ORCID ID 0000-0002-0352-3785

Word count: 4,504 (including tables and footnotes)

Abstract word count: 297 (limit 300)

For peer review only - http://bmjopen.bmj.com/site/about/guidelines.xhtml

ABSTRACT

Objectives: Physical exertion is a high-risk activity for aerosol emission of respiratory pathogens. We aimed to determine the safety and tolerability of healthy young adults wearing different types of facemask during moderate-to-high intensity exercise.

Design: Crossover randomised controlled study, completed between June 2021 and January 2022.

Participants: Volunteers aged 18 to 35 years, who exercised regularly and had no significant pre-existing health conditions..

Interventions: Comparison of wearing a surgical, cloth and FFP3 mask to no mask during four x 15 minute bouts of exercise. Exercise was running outdoors or indoor rowing at moderate-to-high intensity, with consistency of distance travelled between bouts confirmed using a smartphone application (Strava). Each participant completed each bout in random order.

Outcomes: The primary outcome was change in oxygen saturations. Secondary outcomes were change in heart rate, perceived impact of facemask wearing during exercise and willingness to wear a facemask for future exercise.

Results: All 72 volunteers (mean age 23.9) completed the study. Changes in oxygen saturations did not exceed the pre-specified non-inferiority margin (2% difference) with any mask type compared to no mask. At the end of exercise the estimated average difference in oxygen saturations for cloth mask was -0.07% (95%CI -0.39 to 0.25), for surgical 0.28% (-0.04 to 0.60) and for FFP3 -0.21% (-0.53 to 0.11). The corresponding estimated average difference in heart rate for cloth mask was -1.20bpm (-4.56 to 2.15), for surgical 0.36bpm (-3.01 to 3.73) and for FFP3 0.52bpm (-2.85 to 3.89). Wearing a facemask caused additional symptoms such as breathlessness (n=13, 18%) and dizziness (n=7, 10%). 33 participants broadly supported facemask wearing during exercise, particularly indoors, but 22 were opposed.

Conclusion: This study adds to previous findings (mostly from non-randomised studies) that exercising at moderate-to-high intensity wearing a facemask appears to be safe in healthy, young adults.

Trial registration: clinicaltrials.gov, identifier NCT04932226

For peer review only

Strengths and limitations of this study

- This is the largest study to date assessing the safety and tolerability of exercising wearing a facemask.
- The randomised crossover design ensured that the effect of any fatigue from exercise was equal between mask types.
- Some participants exercised outdoors and during cold weather when pulse oximeters were sometimes slow to provide readings, and this may have resulted in a recovery in oxygen levels and an under-estimate of effect size.
- We collected a limited amount of non-invasive physiological data and did not collect data on measures such as end-tidal carbon dioxide or muscle oxygenation.
- We only included healthy young adults who exercised regularly in this study to minimise the risk of serious adverse events, but this may limit the generalisability of our findings to the wider population.

For peer review only

INTRODUCTION

Severe acute respiratory syndrome virus 2 (SARS-CoV-2) is an airborne virus that causes Coronavirus Disease 2019 (COVID-19). Transmission of SARS-CoV-2 is thought to vary based on multiple factors, including host viral load, indoor versus outdoor settings, ambient air flow, volume of speech and physical proximity.[1] A number of these risk factors are relevant to exercise, with previous outbreak clusters reported in boxing or fitness gyms, where people are likely to be talking loudly or breathing heavily in a confined, indoor space.[2, 3] Public health measures such as physical distancing can help reduce transmission risk,[4] but heavy breathing or panting during exercise may still increase the risk of transmitting the SARS-CoV-2 virus, even when people are exercising outdoors.[1]

Facemasks can reduce transmission of SARS-CoV-2 (Box 1). In a recent systematic review and meta-analysis, mask wearing was associated with a decrease in the incidence of COVID-19 (relative risk 0.47, 95%CI 0.29 to 0.75, I^2=84%).[5] However, there has been a lack of clear guidance on wearing facemasks during exercise and a number of recent commentary pieces have suggested that facemasks may interfere with an individual's exercise capacity.[6] Exercise leads to physiological changes, such as an increase in heart and breathing rates, to deliver more oxygen to the body. A facemask may theoretically interfere with gas exchange, potentially leading to an accumulation of carbon dioxide (CO_2) and lactate, with a reduction in oxygen saturation (SaO_2). This may depend in part upon the type of mask worn.[7, 8] Cloth masks tend to capture moisture in exhaled breath to a greater extent than surgical or filtering face piece (FFP) respirator masks, which may particularly interfere with oxygen transfer.[9]

Box 1: Properties of different kinds of face covering

A filtering face piece respirator (FFP) is personal protective equipment made to an industry standard and designed for occupational health use (e.g. healthcare workers treating high-risk infectious patients). FFP3 (equivalent to the US standard N99) filters 99% of airborne particles of the relevant size.

A surgical mask is made of folded paper with waterproof backing. It is designed primarily to block egress (outward emission) of droplets such as saliva or sneezes. Surgical masks are not made to industry standards, nor are they designed to filter airborne particles. Poor

For peer review only - http://bmjopen.bmj.com/site/about/guidelines.xhtml

fit is a commonly-described problem with surgical masks, allowing air to escape through gaps.

The cloth face covering used in this study was a double-layer neck gaiter supplied by Buff©. Cloth face coverings come in many materials and thicknesses; most have not been manufactured to any filtration standard.[10] They are thought to provide partial protection of others by reducing egress of particles and droplets.[11]

The efficacy of masks depends heavily on fit and compliance.[12, 13] Despite their limited efficacy, both surgical masks and cloth face coverings may contribute significantly to reducing SARS-CoV-2 transmission if a high proportion of the population wears them in high-risk settings (e.g. indoors, crowded and at times of high prevalence of Covid-19).[14, 15]

In one laboratory study, a respirator reduced filtration of an artificial respiratory aerosol by 99%; a medical grade ('surgical') mask by 59% and a double neck gaiter by 60%.[16] A modelling study calculated that if two people are both wearing respirators, the risk of infection is reduced to 0.14% of the risk if neither are masked.[13]

There have been a number of recent studies assessing the impact of wearing a FFP or surgical mask during exercise, but most of these included very small numbers of participants.[17, 18] As health and fitness facilities and physical therapy rehabilitation units re-open, it is important to determine to what extent wearing a facemask in these settings is feasible, in addition to other public health measures. We aimed to determine the safety and tolerability of healthy young adults wearing different types of facemask during moderate-to-high intensity exercise.

METHODS

Study design

We conducted a randomised crossover study, where all participants completed four x 15 minute bouts of moderate-to-high intensity exercise wearing three different types of facemask (cloth, surgical or FFP3) and no facemask in a random order, with a 5 minute rest period between each bout of exercise. The study team used an online simple random sequence

For peer review only - http://bmjopen.bmj.com/site/about/guidelines.xhtml

generator to allocate mask order for each participant, without blinding. Participants could state a preference for their choice of exercise, between either running, cycling or rowing.

Participants

People in the community were eligible for the study if they were aged between 18 and 35 years, exercised at least three times a week and were willing and able to give informed consent. We excluded anyone with a significant acute or longstanding medical illness that limited their exercise capacity. Volunteer participants were recruited through student groups at the University of Oxford, Nottingham University and King's College London.

Sample size

When planning the study, there was only limited existing research of changes in SaO_2 during exercise. A 2018 study assessed the effect of aerobic exercise on arterial blood SaO_2 among 36 healthy male volunteers. Mean pre-exercise SaO_2 were 97.3% (standard deviation (SD) 1.3, range 95.0% to 99.0%), compared to mean post-exercise SaO_2 96.2% (2.2, 92.0% to 98.0%).[19] We made a conservative estimate that exercise with a facemask might produce a 1.5% margin of difference in peripheral SaO_2. Based on a standard deviation of 2.6 and power of 80%, we calculated a minimum sample size of 55 people was needed, but aimed to recruit over 60 to allow for some participants dropping out and a 10% margin of error.

Outcomes

The primary outcome was change in SaO_2 between different mask types. Secondary outcomes were change in heart rate during exercise, perceived impact of mask wearing during exercise and willingness to wear a mask for exercise in the future.

Data collection

SaO_2 and heart rate were measured using a finger pulse oximeter immediately prior to first commencing exercise, halfway through, on completion and 1 minute after completion for each bout of exercise. Participants paused their exercise at the midway point of exercise to collect these data. Participants continued to wear their facemask for one minute after completing the exercise, when the final recording for that bout of exercise was collected. Participants were asked to exercise at a consistent intensity between bouts of exercise. To confirm this was achieved, we measured the average speed and distance travelled during each bout of exercise using a Smartphone app (Strava) and assessed these data for significant differences across facemask type.

For peer review only - http://bmjopen.bmj.com/site/about/guidelines.xhtml

During each five-minute rest period the participants stood and completed a study questionnaire. This asked them to rate the comfort, ease of exercise and ease of breathing during exercise with the facemask they had just worn on a ten point Likert scale. At the end of the study session we also asked participants to identify the facemask that they found most difficult to exercise wearing, record any symptoms that they felt during exercise that were different to usual and free text comments regarding their willingness to exercise wearing a mask in the future (see Appendix 1).

Statistical analysis

We calculated the difference in SaO_2 and heart rate (bpm) between exercising with each of the three different facemasks compared to no mask in each participant. We used differences rather than raw outcomes as these are more likely to be normally distributed. Dependency within subjects was accounted for by including a subject specific effect in a two-way Analysis of Variance (ANOVA). Average differences and 95% confidence intervals for the three comparisons (cloth vs none, surgical mask vs none and FFP3 vs none) were calculated using estimated marginal means, averaging over subject. Based on prior literature, we pre-specified a non-inferiority margin of a 2% reduction in SaO_2 and 7bpm increase in heart rate. Non-inferiority was considered demonstrated if the lower bound of the 95% confidence interval did not overlap the 2% margin for SaO_2 and if the upper bound of the 95% confidence interval did not overlap 7 bpm for heart rate. We conducted a sub-group analysis to check for significant changes in SaO_2 or heart rate among participants with a low baseline SaO_2, defined as ≤98%. Comparisons of average distance travelled and ease of breathing whilst wearing different types of facemask compared to no mask were analysed using a Bradley–Terry Model for Paired Preferences.[20] All analyses were done in R version 4.1.1 (2021-08-10)

Qualitative analysis

Free text comments were grouped into categories using a pre-defined framework, based around positive, negative or neutral comments for each type of mask. Using a comment-by-comment coding approach in Excel, we then undertook thematic analysis to identify and group common threads within these categories, seeking emergent as well as anticipated themes.[21, 22]

Patient and public involvement

No patients involved.

For peer review only - http://bmjopen.bmj.com/site/about/guidelines.xhtml

RESULTS

In total, 72 participants completed the study between June 2021 and January 2022, including 33 women (46%) and 39 men (54%). The mean age was 23.9 years (SD 3.55). Most participants exercised 3-4 (n=38, 54%) or 5-6 times per week (n=30, 42%), but 3 participants (4%) exercised every day. One participant was a smoker.

All 72 participants completed all four exercise sessions. One participant completed the exercise on an indoor rowing machine and 71 undertook running outdoors, largely due to restrictions on indoor gatherings and physical distancing that were in place because of the COVID-19 pandemic.

In a small number of participants at each time point it was not possible to obtain either a SaO_2 or heart rate reading on the pulse oximeter and the numbers reported in tables 1, 2 and the supplemental tables reflect this. We recruited beyond our minimum sample size to ensure the study retained sufficient statistical power for our primary analysis.

Oxygen saturations during exercise

Changes in SaO_2 did not exceed the non-inferiority margin at any point of exercise wearing any of the three masks (Table 1). There was a small but statistically significant decrease in SaO_2 during exercise wearing either a cloth mask (estimated average difference (EAD) -0.78%, 95%CI -1.12 to -0.44) or the FFP3 compared to no mask (-0.82%, -1.16 to -0.47), but not when wearing the surgical mask (-0.28%, -0.63 to 0.07). There was no difference in SaO_2 comparing any mask type to no mask immediately after completing exercise. Surgical mask wearing was associated with a small increase in SaO_2 at 1 minute post exercise (0.33%, 0.05 to 0.61).

Table 1. Changes in oxygen saturations comparing different types of facemask worn during exercise

Comparison	Average oxygen saturation (%), with mask	Average oxygen saturation (%), no mask	Estimated average difference (%, 95% confidence interval)	Non-inferior at 2% difference in SaO_2
During exercise (N = 69)				
Cloth - None	96.9	97.7	-0.78 (-1.12 to -0.44)	TRUE

For peer review only - http://bmjopen.bmj.com/site/about/guidelines.xhtml

Comparison	Average oxygen saturation (%), with mask	Average oxygen saturation (%), no mask	Estimated average difference (%, 95% confidence interval)	Non-inferior at 2% difference in SaO_2
Surgical - None	97.4	97.7	-0.28 (-0.63 to 0.07)	TRUE
FFP3 - None	96.9	97.7	-0.82 (-1.16 to -0.47)	TRUE
End of exercise (N = 68)				
Cloth - None	97.0	97.1	-0.07 (-0.39 to 0.25)	TRUE
Surgical - None	97.4	97.1	0.28 (-0.04 to 0.60)	TRUE
FFP3 - None	96.9	97.1	-0.21 (-0.53 to 0.11)	TRUE
1 minute after the end of exercise (N = 67)				
Cloth - None	97.7	97.7	0.04 (-0.25 to 0.32)	TRUE
Surgical - None	98.1	97.7	0.33 (0.05 to 0.61)	TRUE
FFP3 - None	97.9	97.7	0.15 (-0.13 to 0.43)	TRUE

In a sub-group analysis among those with lower baseline SaO_2, changes in SaO_2 still did not exceed the non-inferiority margin of 2% although SaO_2 during exercise was 1% lower among people wearing either the FFP3 (EAD -0.88, 95%CI -1.47 to -0.29) or cloth mask (-0.90, -1.48 to -0.32) (Supplemental table 1a). Even those with SaO_2 >98% at baseline had a small but statistically significant decrease in SaO_2 during exercise when wearing either the cloth or FFP3 mask (Supplemental table 1b).

Changes in heart rate, performance or additional symptoms during exercise

There was no difference in heart rate at any time point comparing the three types of mask to no mask (Table 2) and changes in heart rate did not exceed the non-inferiority margin.

Table 2. Changes in heart rate comparing different types of facemask worn during exercise

Comparison	Average heart rate (bpm), with mask	Average heart rate (bpm), no mask	Estimated average difference (bpm, 95% confidence interval)	Non-inferior @ +7bpm
During exercise (N = 69)				

For peer review only - http://bmjopen.bmj.com/site/about/guidelines.xhtml

Comparison	Average heart rate (bpm), with mask	Average heart rate (bpm), no mask	Estimated average difference (bpm, 95% confidence interval)	Non-inferior @ +7bpm
Cloth - None	157.5	157.3	0.10 (-2.90 to 3.10)	TRUE
Surgical - None	157.8	157.3	0.78 (-2.33 to 3.89)	TRUE
FFP3 - None	159.9	157.3	2.84 (-0.24 to 5.91)	TRUE
End of exercise (N = 69)				
Cloth - None	156.9	158.2	-1.20 (-4.56 to 2.15)	TRUE
Surgical - None	158.7	158.2	0.36 (-3.01 to 3.73)	TRUE
FFP3 - None	158.9	158.2	0.52 (-2.85 to 3.89)	TRUE
1 minute after the end of exercise (N = 67)				
Cloth - None	124.8	124.2	0.40 (-2.15 to 2.95)	TRUE
Surgical - None	124.6	124.2	0.49 (-2.03 to 3.02)	TRUE
FFP3 - None	125.3	124.2	1.45 (-1.11 to 4.01)	TRUE

Among the subgroups with $SaO_2 \leq 98\%$ or $SaO_2 > 98\%$ at baseline, there was no significant difference in average heart rate during or after exercise with any facemask (Supplemental tables 2a and 2b). However, for the subgroup with $SaO_2 \leq 98\%$ the upper 95% CI did exceed the non-inferiority margin of 7bpm for the FFP3 mask compared to no mask at each time point measured, although the sample size for this analysis was small. This was also true for the surgical mask at the end of exercise and after a 1 minute rest period (Supplemental table 2a). For the subgroup with $SaO_2 > 98\%$ the upper 95% CI exceeded the non-inferiority margin for the FFP3 and surgical masks compared to no mask during exercise only (Supplemental table 2b).

A post-hoc sensitivity analysis excluding the one person who rowed rather than ran did not significantly alter the results for change in SaO_2 or heart rate (Supplemental tables 3a and b).

For peer review only - http://bmjopen.bmj.com/site/about/guidelines.xhtml

Across participants there was no significant difference in average speed or distance travelled when comparing any of the facemasks to no mask, confirming participants had been able to exercise at a fairly consistent speed between bouts of exercise (Table 3).

Table 3. Comparison of average distance travelled and speed whilst wearing different types of facemask compared to no mask

Comparison	Average distance travelled (kilometres) with mask	Average distance travelled (kilometres), no mask	Estimated average difference in distance (km, 95% confidence interval)	Average speed (km/hour) with mask	Average speed (km/hour), no mask	Estimated average difference in speed (km/h, 95% confidence interval)
Distance travelled (km) during exercise (N = 71)						
Cloth mask - No facemask	2.75	2.82	-0.07 (-0.15 to 0.01)	11.06	11.37	-0.31 (-0.64 to 0.02)
Surgical mask - No facemask	2.78	2.82	-0.04 (-0.12 to 0.04)	11.27	11.37	-0.10 (-0.43 to 0.22)
FFP3 - No facemask	2.76	2.82	-0.06 (-0.14 to 0.02).	11.12	11.37	-0.24 (-0.57 to 0.08)

The most common symptoms participants reported feeling wearing a facemask during exercise were increased breathlessness (n=13, 18%), dizziness (n=7, 10%), 'other' symptoms (n=7, 10%) and fatigue (n=6, 8%). The 'other' symptoms recorded as free-text comments were feeling light-headed, facial discomfort, skin irritation, a runny nose, pressure in the eyes, cramping and a stitch. Four participants found wearing a mask claustrophobic and one developed a headache. No participants reported feeling anxious or drowsy and none had to stop during exercise due to side-effects.

Perception of comfort, breathing and ease of exercise

When asked which facemask they found most difficult to exercise wearing, 40 (56%) participants said the cloth mask, 27 (38%) said FFP3 and 4 (6%) said the surgical mask.

For peer review only - http://bmjopen.bmj.com/site/about/guidelines.xhtml

Participants felt that the surgical mask was the most comfortable (Figure 1) and the easiest to breathe through (Figure 2). In a direct comparison, the probability of a surgical mask being ranked easier to exercise in than a cloth mask was 0.81 (95%CI 0.72 to 0.88, p=0.00) and being easier than FFP3 was 0.80 (0.73 to 0.86, p=0.00) (Table 4). Surgical masks were also ranked easier to breathe through than the cloth mask (probability 0.86, 95%CI 0.78 to 0.91) or FFP3 (0.83, 0.76 to 0.88). The FFP3 and cloth mask were ranked equal for ease of exercise and breathing through.

Table 4. Comparison of reported comfort, ease of breathing and ease of overall exercise between the different types of facemask worn during exercise

Type 1	Type 2	Number rating Type 1 lower	Number rating Type 2 lower	Probability type 1 rated lower (95% confidence interval)	P-value
Ease of Exercise, lower score = easier					
Surgical[1]	FFP3	51	12	0.80 (0.73 to 0.86)	0.00
Surgical	Cloth	50	12	0.81 (0.72 to 0.88)	0.00
Cloth	FFP3[2]	29	31	0.49 (0.38 to 0.60)	0.84
Ease of Breathing, lower score = easier					
Surgical[1]	FFP3	55	11	0.83 (0.76 to 0.88)	0.00
Surgical	Cloth	59	10	0.86 (0.78 to 0.91)	0.00
Cloth	FFP3[2]	28	36	0.44 (0.34 to 0.55)	0.30

[1]Surgical is ranked 1st

[2] FFP3 and cloth are equal 2nd

In free text comments (Supplemental table 4), participants commented on the cloth mask becoming *'soaked through with sweat and water vapour'*. A number commented on how the cloth mask '*sucks in when breathing in'* or '*kept getting in my mouth'*, whilst others found it '*kept slipping down'*. One participant described running in the cloth mask as '*like being waterboarded'*. There were 35 negative free text comments about the cloth mask and one positive comment.

For peer review only - http://bmjopen.bmj.com/site/about/guidelines.xhtml

There were 15 negative free text comments about the FFP3 mask, which some participants found '*constraining*', '*so tight fitting it felt difficult to breathe'* and caused '*nose pain'*. Some people found this made them feel '*a bit dizzy'*, '*really lightheaded'*, or '*as though my heart was going really high when I was wearing it*'. However, there were also 10 positive comments about wearing the FFP3, with these participants reporting '*a good fit'*, which '*was very comfortable'*, '*amenable to glasses-wearing'* and which '*didn't shake at all whilst running, unlike the others*'.

There were 11 positive comments about wearing the surgical mask, mostly reporting it was '*fine*', the *'easiest one'* or caused '*little restriction'*. The four negative comments about the surgical mask focused on glasses steaming up or the mask slipping.

Willingness to wear a facemask for exercise in the future

There were 33 participants who were accepting of the idea of mandatory facemasks, 22 who were opposed and 17 who gave more neutral responses (Supplemental table 5). Positive replies included '*It would make no real impact to my exercise having to wear a facemask'*, '*Wouldn't bother me too much'* and '*To be honest, if wearing a facemask during exercise will limit the spread of COVID then I am happy to do so'*. A number of participants mentioned they '*wouldn't mind doing so indoors'*, or '*only if it was the surgical mask'*. In the neutral group there were comments such as '*I would tolerate it but not advocate it'* and '*I would if I had to'*. Among those opposed people mentioned '*I would be annoyed, because I don't think that it's really needed for preventing transmission, especially outdoors'* and that they '*would rather have a choice in the matter'*. Some people compared favourably the experience of exercising with no mask compared to wearing any mask, with one saying '*When I ran with no mask at the end it felt like breathing in HD'* (meaning high definition).

DISCUSSION

We found no clinically significant reduction in SaO_2 or heart rate with any of the three facemasks, suggesting that exercising at moderate-to-high intensity wearing a facemask appears to be safe in healthy, young adults. However, around one in three participants reported additional symptoms when wearing a facemask, including feeling more breathless, dizzy or fatigued. Surgical facemasks were experienced as the most comfortable, easiest to breathe through and had the least impact on exercise, though importantly we did not measure whether these masks were well-fitting and poor fit of surgical masks is common and greatly

For peer review only - http://bmjopen.bmj.com/site/about/guidelines.xhtml

reduces protection.[13] There was most support for wearing a surgical facemask during indoor exercise if needed to reduce the spread of COVID-19, though around a third of participants were opposed to mandatory wearing of facemasks during exercise.

Strengths and limitations

To our knowledge, this is the largest study to date to investigate the safety and tolerability of wearing a facemask during exercise. We used a randomised crossover design to ensure that the effect of any fatigue from exercise was equal between mask types. We asked participants to exercise at a consistent speed between study sessions and recorded their average speed and distance travelled, with no significant difference in exercise performance based on type of facemask worn.

We chose to recruit only healthy young participants to reduce the risk of serious adverse events, because of the limited pre-existing data demonstrating wearing a facemask during exercise was safe. However, our results may not be generalisable to people who have underlying health conditions or restricted exercise capacity and further research is needed in these populations.

We collected a limited amount of non-invasive physiological data from participants. Healthy young participants may maintain a constant SaO_2 and heart rate through compensatory mechanisms, such as increased respiratory rate, that we have not captured in our analysis. However, all of our participants were able to complete the exercise wearing each mask type with no significant change in their speed or distance travelled, supporting the finding that healthy young people can safely exercise wearing facemasks.

Because of the COVID-19 pandemic and restrictions in social interaction, we conducted almost all of the study sessions outdoors. Study sessions took place across a range of seasons. During cold weather some participants had cold peripheries whilst exercising, meaning the pulse oximeters were not always able to record SaO_2 and heart rate or were slow to do so. Delays in obtaining accurate oximeter readings post-exercise may have led to some recovery in heart rate and SaO_2 that would result in an under-estimate of any change in these parameters. For these reasons we also asked participants to fit their own facemasks and we did not perform fitting tests. Whilst this may have led to some discrepancy in mask fit between participants, it does reflect how facemasks would be worn in practice. We had

For peer review only - http://bmjopen.bmj.com/site/about/guidelines.xhtml

originally proposed to use FFP2 masks in our study protocol but there were supply issues that impacted us ordering these and we changed to use FFP3 masks instead.

Comparison with other studies

Our results support the findings of a recent systematic review and meta-analysis, which assessed the impact of wearing a facemask during exercise.[23] This reported no significant difference in exercise performance among studies of surgical facemasks (standard mean difference (SMD) -0.05, 95CI -0.16 to 0.07) or N95 masks (-0.16, -0.54 to 0.22).[23] Patients did have increased ratings of perceived exertion (0.33, 0.09 to 0.58) and dyspnoea (0.60, 0.30 to 0.90) with all masks.[23] In contrast to this review, we include cloth masks in the comparison of facemasks types. The exercise tested in these studies was heterogeneous, ranging from 1 hour treadmill walks at low intensity, to strength training with half-squats and progressive exercise training on a bicycle. Our results demonstrate the principal findings of this review are applicable to healthy young adults during moderate-to-high intensity exercise.

A second recent systematic review of facemask wearing during rest, work or exercise identified 14 crossover trials with 25 independent intervention arms.[24] There were a total of 246 participants across these studies. High intensity exercise was associated with a small reduction in SaO_2 (SMD -0.40, 95%CI -0.70 to – 0.09) and lower respiratory rates (-0.25, -0.44 to -0.06), particularly when wearing a FFP2 or N95 facemask.[24] However, there was no association with mask wearing and change in exercise performance, heart rate or exhaled CO_2. This review suggests that undertaking exhausting exercise wearing FFP-type facemasks may reduce SaO_2 but supports our finding that mask wearing appears to be safe and does not negatively affect overall performance.

Implications for policy and research

Governments globally are having to decide which public health measures to continue in response to changing incidence of COVID-19. Interventions that can contribute towards reducing transmission, whilst allowing people to return to normal life, are particularly important. Facemasks can reduce transmission of the SARS-CoV-2 virus and appear to be safe to wear among young, healthy adults during exercise. Public health messaging could support their use in higher risk settings, such as indoor settings with poor ventilation or outdoor exercise among large volumes of people. Surgical masks appear to be the least restrictive type of mask to wear and may help reduce transmission by preventing the forward

For peer review only - http://bmjopen.bmj.com/site/about/guidelines.xhtml

propulsion of a large jet of exhaled aerosol particles. Cloth masks and the FFP3 were ranked similarly by participants in our study in terms of ease of exercise and breathing through. This suggests that policy makers could consider recommending either surgical masks or the more secure FFP3, as opposed to cloth masks, should facemasks be required during exercise. However, the comparative effectiveness of different mask types in reducing the risk of transmission of SARS-CoV-2 does remains uncertain and could be a focus for future research. Future research could also examine the safety and tolerability of wearing a facemask during exercise among people with underlying health conditions, as results may differ from our healthy population. For example, a previous study of 97 people with chronic obstructive pulmonary disease performing a 6-minute walking test wearing an N95 mask reported that those with more significant disease were more likely to experience dyspnoea or breathing discomfort.[25]

Wearing a facemask during exercise does not negatively impact exercise performance. It is possible there may even be benefits for longer-term endurance exercise performance if facemask wearing did induce mild hypoxia. Hypoxic exercise training has previously been reported to improve cardiac haemodynamics and exercise capacity,[26, 27] though these studies typically recruited only small numbers of participants and have not been consistently replicated.[28] Further research into the long-term impact on exercise performance of wearing a facemask during exercise could help better inform athletes of the relative risks and benefits of this intervention.

CONCLUSION

In this randomised crossover trial we found that healthy young adults were able to exercise at moderate-to-high intensity wearing either a surgical, cloth or FFP facemask without significant restriction on exercise performance or physiological limitation. However, wearing a facemask during exercise made some participants feel more breathless, dizzy or fatigued and most people felt it was less comfortable and enjoyable than exercising without a mask. There was limited support for wearing a facemask to exercise across all settings, but these results could support the use of facemask wearing whilst exercising in a limited number of high-risk settings as a safe intervention to reduce the risk of SARS-CoV-2 transmission.

For peer review only - http://bmjopen.bmj.com/site/about/guidelines.xhtml

Contributorship

NRJ and TG conceived the study. NRJ, TG and HPD wrote the protocol. NRJ, SM, KN and JB conducted all study visits. JO and NRJ carried out the analysis. NRJ wrote the first draft of the manuscript and is the guarantor and corresponding author. RH served as a scientific advisor. All authors contributed to the interpretation of the results and revisions of the manuscript. The corresponding author attests that all listed authors meet authorship criteria and that no others meeting the criteria have been omitted.

Funding Statement

Buff© provided 60 original tubular cotton neckwear free of charge, which were used as the cloth masks. Buff© had no role in study design, data collection, data analysis, data interpretation, writing of the report, or any other aspect of the study.

NRJ is supported by a Wellcome Trust Doctoral Research Fellowship (203921/Z/16/Z). FDRH acknowledges his part-funding from the NIHR Applied Research Collaboration (ARC) Oxford Thames Valley, the NIHR Oxford Biomedical Research Centre (BRC), and the NIHR Oxford Medtech and In-Vitro Diagnostics Co-operative. TG's research is supported by Oxford NIHR Biomedical Research Centre (grant no BRC-1215-20008).

Competing interests

No competing interests.

Ethics approval

The University of Oxford, Central University Research Ethics Committee provided ethical approval for the study (reference R&2015/RE001).

Data sharing

To request access to deidentified participant data or the study protocol, please contact the lead author.

For peer review only - http://bmjopen.bmj.com/site/about/guidelines.xhtml

Registration

The trail was registered in advance on ClinicalTrials.gov on 21st June 2021, 'Face Masks and Oxygen Levels – What Happens During Exercise? (Masks in Exercise – a Randomised Trial) (MERIT). The NCT number is NCT04932226.

Acknowledgements

Thanks to Robert Temple who also helped with data collection on one study visit.

For peer review only

REFRENCES

1. Jones, N.R., et al., *Two metres or one: what is the evidence for physical distancing in covid-19?* BMJ, 2020. **370**: p. m3223.
2. Chu, D.K.W., et al., *SARS-CoV-2 Superspread in Fitness Center, Hong Kong, China, March 2021.* Emerg Infect Dis, 2021. **27**(8): p. 2230-2232.
3. Jang, S., S.H. Han, and J.Y. Rhee, *Cluster of Coronavirus Disease Associated with Fitness Dance Classes, South Korea.* Emerg Infect Dis, 2020. **26**(8): p. 1917-1920.
4. Helsingen, L.M., et al., *Covid-19 transmission in fitness centers in Norway - a randomized trial.* BMC Public Health, 2021. **21**(1): p. 2103.
5. Talic, S., et al., *Effectiveness of public health measures in reducing the incidence of covid-19, SARS-CoV-2 transmission, and covid-19 mortality: systematic review and meta-analysis.* BMJ, 2021. **375**: p. e068302.
6. Bottoms, L. *Why it could be dangerous to exercise with a face mask on*. 2020 05.08.2020]; Available from: https://theconversation.com/why-it-could-be-dangerous-to-exercise-with-a-face-mask-on-140277.
7. Li, Y., et al., *Effects of wearing N95 and surgical facemasks on heart rate, thermal stress and subjective sensations.* Int Arch Occup Environ Health, 2005. **78**(6): p. 501-9.
8. Person, E., et al., *[Effect of a surgical mask on six minute walking distance].* Rev Mal Respir, 2018. **35**(3): p. 264-268.
9. MacIntyre, C.R., et al., *A cluster randomised trial of cloth masks compared with medical masks in healthcare workers.* BMJ Open, 2015. **5**(4): p. e006577.
10. Konda, A., et al., *Aerosol Filtration Efficiency of Common Fabrics Used in Respiratory Cloth Masks.* ACS Nano, 2020. **14**(5): p. 6339-6347.
11. Clase, C.M., et al., *Forgotten Technology in the COVID-19 Pandemic: Filtration Properties of Cloth and Cloth Masks-A Narrative Review.* Mayo Clin Proc, 2020. **95**(10): p. 2204-2224.
12. Czypionka, T., et al., *Masks and Face Coverings for the Lay Public : A Narrative Update.* Ann Intern Med, 2021. **174**(4): p. 511-520.
13. Bagheri, G., et al., *An upper bound on one-to-one exposure to infectious human respiratory particles.* Proc Natl Acad Sci U S A, 2021. **118**(49).
14. Cheng, Y., et al., *Face masks effectively limit the probability of SARS-CoV-2 transmission.* Science, 2021.
15. Ku, D., et al., *Safe traveling in public transport amid COVID-19.* Sci Adv, 2021. **7**(43): p. eabg3691.
16. Lindsley, W.G., et al., *Efficacy of universal masking for source control and personal protection from simulated cough and exhaled aerosols in a room.* J Occup Environ Hyg, 2021. **18**(8): p. 409-422.
17. Mapelli, M., et al., *"You can leave your mask on": effects on cardiopulmonary parameters of different airway protective masks at rest and during maximal exercise.* Eur Respir J, 2021. **58**(3).
18. Kim, J.H., S.M. Benson, and R.J. Roberge, *Pulmonary and heart rate responses to wearing N95 filtering facepiece respirators.* Am J Infect Control, 2013. **41**(1): p. 24-7.
19. Eroğlu, H., B. Okyaz, and U. Türkçapar, *The Effect of Acute Aerobical Exercise on Arterial Blood Oxygen Saturation of Athletes.* Journal of Education and Training Studies, 2018. **6**: p. 74-79.
20. Turner, H. and D. Firth, *Bradley-Terry Models in R: The BradleyTerry2 Package.* Journal of Statistical Software, 2012. **48**(9): p. 1–21.
21. Braun, V. and V. Clarke, *Using thematic analysis in psychology.* Qualitative Research in Psychology, 2006. **3**(2): p. 77-101.

For peer review only - http://bmjopen.bmj.com/site/about/guidelines.xhtml

22. Ziebland, S. and A. McPherson, *Making sense of qualitative data analysis: an introduction with illustrations from DIPEx (personal experiences of health and illness).* Med Educ, 2006. **40**(5): p. 405-14.
23. Shaw, K.A., et al., *The impact of face masks on performance and physiological outcomes during exercise: a systematic review and meta-analysis.* Appl Physiol Nutr Metab, 2021. **46**(7): p. 693-703.
24. Engeroff, T., D.A. Groneberg, and D. Niederer, *The Impact of Ubiquitous Face Masks and Filtering Face Piece Application During Rest, Work and Exercise on Gas Exchange, Pulmonary Function and Physical Performance: A Systematic Review with Meta-analysis.* Sports Med Open, 2021. **7**(1): p. 92.
25. Kyung, S.Y., et al., *Risks of N95 Face Mask Use in Subjects With COPD.* Respir Care, 2020. **65**(5): p. 658-664.
26. Chacaroun, S., et al., *Hypoxic Exercise Training to Improve Exercise Capacity in Obese Individuals.* Med Sci Sports Exerc, 2020. **52**(8): p. 1641-1649.
27. Wang, J.S., et al., *Hypoxic exercise training improves cardiac/muscular hemodynamics and is associated with modulated circulating progenitor cells in sedentary men.* Int J Cardiol, 2014. **170**(3): p. 315-23.
28. Vogt, M. and H. Hoppeler, *Is hypoxia training good for muscles and exercise performance?* Prog Cardiovasc Dis, 2010. **52**(6): p. 525-33.

For peer review only

For peer review only - http://bmjopen.bmj.com/site/about/guidelines.xhtml

Figure legends

Figure 1. Reported comfort of wearing different types of facemask during exercise

Caption: The graph plots the number of participants who reported each comfort score, between 1 and 9, for each of the three different types of facemask or no mask.

Figure 2. Reported ease of breathing wearing different types of facemask during exercise

Caption: The graph plots the number of participants who reported each ease of breathing score, between 1 and 9, for each of the three different types of facemask or no mask.

For peer review only

Figure 1. Reported comfort of wearing different types of facemask during exercise

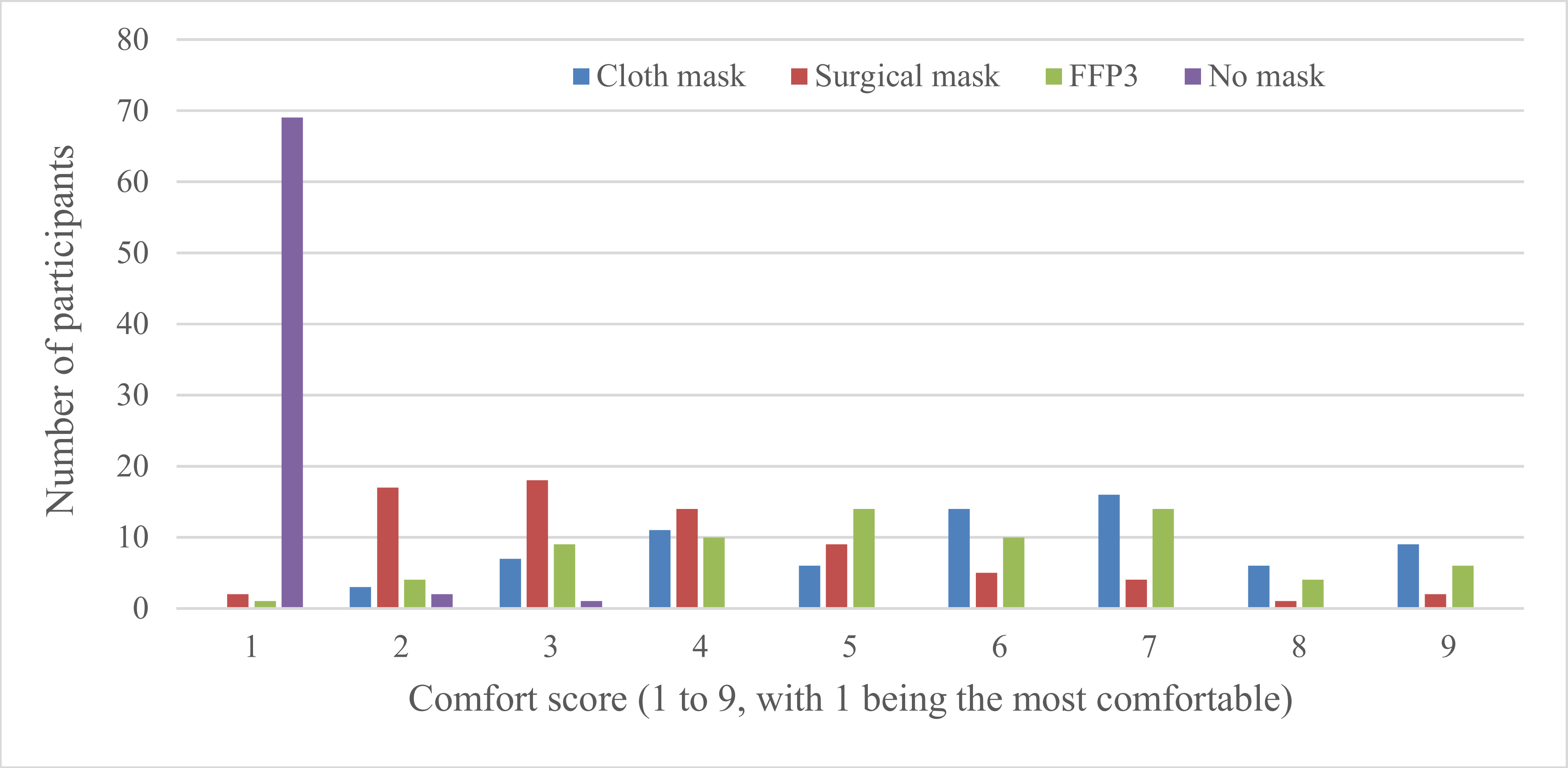

Figure 2. Reported ease of breathing wearing different types of facemask during exercise

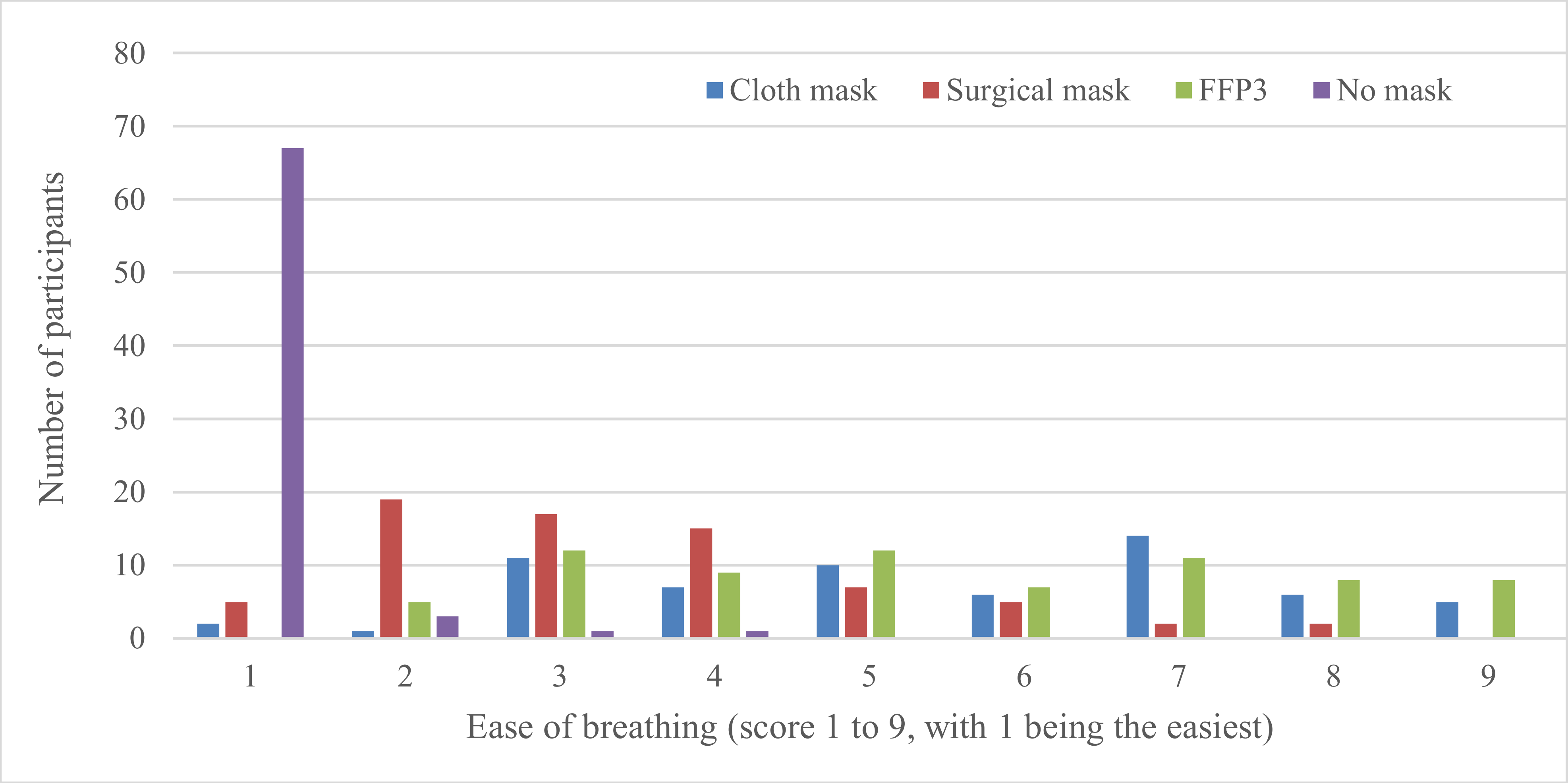

Supplemental Table 1a. Changes in oxygen saturations comparing different mask types worn during exercise. Sub-group analysis of participants with resting oxygen saturations of ≤ 98%

Comparison	Average oxygen saturations (%) with mask	Average oxygen saturations (%), no mask	Estimated average difference (%, 95% confidence interval)	Non-inferior @ -2%
During exercise (N = 30)				
Cloth - None	96.5	97.4	-0.90 (-1.48 to -0.32)	TRUE
Surgical - None	97.3	97.4	-0.12 (-0.72 to 0.49)	TRUE
FFP3 - None	96.5	97.4	-0.88 (-1.47 to -0.29)	TRUE
End of exercise (N = 29)				
Cloth - None	96.9	97.0	-0.07 (-0.57 to 0.43)	TRUE
Surgical - None	97.0	97.0	-0.03 (-0.55 to 0.49)	TRUE
FFP3 - None	97.0	97.0	0.00 (-0.52 to 0.52)	TRUE
1 minute after the end of exercise (N = 30)				
Cloth - None	97.6	97.7	-0.11 (-0.61 to 0.39)	TRUE
Surgical - None	97.9	97.7	0.18 (-0.32 to 0.67)	TRUE
FFP3 - None	97.6	97.7	-0.08 (-0.57 to 0.42)	TRUE

For peer review only - http://bmjopen.bmj.com/site/about/guidelines.xhtml

Supplemental Table 1b. Changes in oxygen saturations comparing different mask types worn during exercise. Sub-group analysis of participants with resting oxygen saturations of > 98%

Comparison	Average oxygen saturations (%) with mask	Average oxygen saturations (%), no mask	Estimated average difference (%, 95% confidence interval)	Non-inferior @ -2%
During exercise (N = 39)				
Cloth - None	97.3	97.9	-0.69 (-1.11 to -0.27)	TRUE
Surgical - None	97.5	97.9	-0.40 (-0.83 to 0.03)	TRUE
FFP3 - None	97.2	97.9	-0.77 (-1.19 to -0.35)	TRUE
End of exercise (N = 39)				
Cloth - None	97.2	97.2	-0.07 (-0.48 to 0.35)	TRUE
Surgical - None	97.7	97.2	0.51 (0.11 to 0.92)	TRUE
FFP3 - None	96.9	97.2	-0.36 (-0.76 to 0.05)	TRUE
1 minute after the end of exercise (N = 37)				
Cloth - None	97.8	97.7	0.16 (-0.18 to 0.49)	TRUE
Surgical - None	98.2	97.7	0.46 (0.13 to 0.79)	TRUE
FFP3 - None	98.1	97.7	0.32 (-0.01 to 0.66)	TRUE

Supplemental Table 2a. Changes in heart rate comparing different mask types worn during exercise. Sub-group analysis of participants with resting oxygen saturations of ≤ 98%

Contrast	Average heart rate (%) with mask (beats per minute)	Average heart rate (%), no mask (beats per minute)	Estimated average difference (bpm, 95% confidence interval)	Non-inferior @ +7bpm
During exercise (N = 29)				
Cloth - None	156.4	158.0	-1.69 (-6.55 to 3.17)	TRUE
Surgical - None	155.9	158.0	-1.41 (-6.57 to 3.75)	TRUE
FFP3 - None	159.5	158.0	2.21 (-2.95 to 7.37)	FALSE
End of exercise (N = 29)				
Cloth - None	151.5	155.9	-4.17 (-10.89 to 2.54)	TRUE
Surgical - None	156.5	155.9	0.36 (-6.59 to 7.31)	FALSE
FFP3 - None	158.4	155.9	2.04 (-4.91 to 8.99)	FALSE
1 minute after the end of exercise (N = 29)				
Cloth - None	123.5	123.2	0.15 (-4.47 to 4.77)	TRUE
Surgical - None	125.4	123.2	2.74 (-1.88 to 7.36)	FALSE
FFP3 - None	125.7	123.2	2.82 (-1.96 to 7.60)	FALSE

Supplemental Table 2b. Changes in heart rate comparing different mask types worn during exercise. Sub-group analysis of participants with resting oxygen saturations of > 98%

Contrast	Average heart rate (%) with mask (beats per minute)	Average heart rate (%), no mask (beats per minute)	Estimated average difference (bpm, 95% confidence interval)	Non-inferior @ +7bpm
During exercise (N = 39)				
Cloth - None	158.4	156.8	1.59 (-2.22 to 5.40)	TRUE
Surgical - None	160.1	156.8	3.39 (-0.49 to 7.27)	FALSE
FFP3 - None	161.6	156.8	4.74 (0.93 to 8.55)	FALSE
End of exercise (N = 39)				
Cloth - None	162.2	159.9	2.11 (-1.03 to 5.26)	TRUE
Surgical - None	160.5	159.9	0.54 (-2.54 to 3.62)	TRUE
FFP3 - None	159.8	159.9	-0.18 (-3.26 to 2.90)	TRUE
1 minute after the end of exercise (N = 37)				
Cloth - None	126.2	125.3	0.71 (-2.31 to 3.73)	TRUE
Surgical - None	124.5	125.3	-0.97 (-3.93 to 1.99)	TRUE
FFP3 - None	125.7	125.3	0.73 (-2.23 to 3.69)	TRUE

Supplemental Table 3a. Changes in oxygen saturations comparing different mask types worn during exercise. Sensitivity analysis excluding the one participant who completed the exercise rowing instead of running

Comparison	Average oxygen saturations (%) with mask	Average oxygen saturations (%), no mask	Estimated average difference (%, 95% confidence interval)	Non-inferior @ -2%
During exercise (N = 68)				
Cloth - None	97.0	97.7	-0.75 (-1.09 to -0.41)	TRUE
Surgical - None	97.5	97.7	-0.27 (-0.63 to 0.08)	TRUE
FFP3 - None	96.9	97.7	-0.81 (-1.16 to -0.47)	TRUE
End of exercise (N = 67)				
Cloth - None	97.1	97.1	-0.05 (-0.38 to 0.27)	TRUE
Surgical - None	97.4	97.1	0.30 (-0.02 to 0.63)	TRUE
FFP3 - None	96.9	97.1	-0.21 (-0.54 to 0.11)	TRUE
1 minute after the end of exercise (N = 66)				
Cloth - None	97.7	97.7	0.05 (-0.24 to 0.34)	TRUE
Surgical - None	98.1	97.7	0.35 (0.07 to 0.64)	TRUE
FFP3 - None	97.9	97.7	0.16 (-0.12 to 0.45)	TRUE

Supplemental Table 3b. Changes in heart rate comparing different mask types worn during exercise. Sensitivity analysis excluding the one participant who completed the exercise rowing instead of running

Contrast	Average heart rate (%) with mask (beats per minute)	Average heart rate (%), no mask (beats per minute)	Estimated average difference (bpm, 95% confidence interval)	Non-inferior @ +7bpm
During exercise (N = 68)				
Cloth - None	157.6	157.4	0.09 (-2.95 to 3.13)	TRUE
Surgical - None	157.8	157.4	0.67 (-2.49 to 3.83)	FALSE
FFP3 - None	160.0	157.4	2.85 (-0.27 to 5.97)	FALSE
End of exercise (N = 68)				
Cloth - None	157.0	158.6	-1.58 (-4.98 to 1.83)	TRUE
Surgical - None	158.8	158.6	0.07 (-3.35 to 3.49)	TRUE
FFP3 - None	159.1	158.6	0.22 (-3.20 to 3.64)	TRUE
1 minute after the end of exercise (N = 66)				
Cloth - None	124.9	124.3	0.50 (-2.07 to 3.07)	TRUE
Surgical - None	124.7	124.3	0.55 (-1.99 to 3.09)	TRUE
FFP3 - None	125.7	124.3	1.81 (-0.77 to 4.39)	TRUE

Supplemental table 4. Participants free text comments regarding the ease of exercising wearing different mask types. These have been categorised as either positive, negative or neutral comments and by facemask type.

Positive comments	Neutral comments	Negative comments
**Any mask**		
It wasn't actually that much worse than exercising with no mask and it did keep my face warm on a very cold day.	Inconvenient but bearable	Considerably more difficult than normal
Didn't find that any mask slowed me down.	As the exercise went on it became easier to breathe with a mask on	With all of them I didn't actually become short of breath it was more discomfort. With the cloth one and the surgical one slightly the main issue was due to them being flexible the fabric would go into my mouth when breathing in.
	It is relevant whether the mask is stable	Masks get in the way, maybe not on the actual performance. Placebo effect
	It was slightly more uncomfortable than not wearing a mask, but not too bad.	All of the masks made my face extra sweaty
	All were warmer than no mask	I'd prefer not to exercise with a mask. There was a lot of humidity with the tighter fitting masks and it makes you much more aware of your breathing.
		The buff and surgical masks were similar - not too bad but a lot worse than no mask. It was harder across the 15 minutes with condensation from the mask. You feel you overheat and there is steaming up of your glasses
		The humidity was unpleasant rather than being difficult to breathe. I found it harder to breathe through my mouth wearing any of the masks.
		It wasn't that much more difficult wearing a mask but the sweatiness was unpleasant
		Surprised to cover roughly the same distance each time even when it more difficult in some of the masks than others - the cloth and FFR.
		the FFP and cloth mask were much worse
**Cloth**		
Cloth was good for me as I wear glasses and allowed me to breathe through my nose and mouth more easily		Cloth mask was surprisingly uncomfortable - about as bad as wearing the FFP2 mask. Noticed that the cloth mask quickly became soaked through with sweat and water vapour which made it very difficult to breathe through the nose.

For peer review only

The cloth mask was particularly uncomfortable as it clung to my face and was very distracting every time you breathe. This was a particular problem as I was breathing through my mouth during the exercises

None were great. The cloth had the added disadvantage of making the rest of your face hot

Cloth mask gets very moist and gets inhaled and falls a lot. Breathing pattern altered with masks - not breathing in, out, in, out but more in, in, out, in, in, out...

Cloth mask felt quite claustrophobic

Cloth mask tight and hot around face

Cloth mask got wet

Cloth mask is distracting (slips a lot).

It was hard to breathe through my mouth with the cloth mask

Wearing cloth mask was hardest because the cloth became humid and block air from entering and exiting mouth.

Cloth mask is the worst to wear when I do exercise

Cloth mask so very hard to breathe in

The cloth mask was annoying because it kept slipping down.

Cloth face mask kept falling down

Cloth mask kept going in my mouth

Running with the mask fogged up my glass, which was annoying. This was worst with the cloth mask

The buff cloth masked pressed down on my nose, which meant I had to breathe through my nose (not a good thing).

Dont make people wear a cloth mask, it sucks in when breathing and it's difficult to breathe.

The cloth mask kept falling and was terrible.

The buff mask was worse as it would suck into your mouth.

I was surprised that I found the cloth mask harder

Cloth was uncomfortable due to the stickiness

Cloth mask felt very close and humid.

Cloth mask was the worst, got very wet, like being waterboarded, sweaty, incompatible with glasses.

For peer review only

		Cloth mask kept slipping down and being sucked in when breathing in
		Didn't like cloth mask - too hot, too close to mouth, claustrophobic
		Buff was generally horrible
		Cloth mask didn't fit well with glasses, kept slipping. Cloth mask was very sticky, stuck straight to nose and mouth, really uncomfortable, no gap to breathe.
		Cloth mask kept getting in my mouth, couldn't out of nose well.
		Cloth mask kept going in my mouth.
		FFR better than cloth more room to breathe
		The buff was claustrophobic
		Cloth mask was hardest, kept sliding down, and going in to mouth when breathing.
		Cloth kept sliding down and made face hot but otherwise didn't mind.
		Cloth gets in mouth when breathing in, kept having to pull it up.
**FFP3**		
I was surprised the FFR was easier than the others - it helped that it doesn't move around much	Exercising in the FFR wasn't as bad as I thought it'd be	The FFR was extremely difficult to exercise with compared to the rest - I was out of breath constantly and I had to slow down or switch to walking just to catch my breath. While wearing the FFR mask, I also felt a lot of strain (pressure) in my eyes.
FFR was very comfortable		Got nose pain from metal on resp mask
The FFR was a good fit		FFR mask is constraining.
Found FFR similar to the surgical mask - both were fine		It was hard to breathe through my nose with the FFR mask.
Liked the FFP - less annoying, better than expected, didn't move, didn't interfere with glasses.		Felt dizzy with FFR mask
FFR easiest to breathe in		The FFR was definitely the worst, it was so tight fitting it felt difficult to breathe
Found FFR to be the same as normal		The FFR mask was very tight fitting and it felt as though my heart rate was going very high when I was wearing it.
FFR didn't shake at all whilst running unlike the others. Didn't really notice I was wearing the FFR mask, although got quite		It felt like the FFR restricted your breathing

warm		
FFR was amenable to glasses-wearing, the fixed gap made it easier to breathe, found it fine in general.		FFR worse than surgical.
I felt close to normal in the FFP		FFR wasn't as bad as I thought it'd be, although still quite bad and the worse one for me. I found the shape around the nose made it more difficult to breathe
		Felt really lightheaded and struggled to breathe when wearing FFR, really uncomfortable.
		felt a bit dizzy wearing FFR
		FFR sweaty, made face really hot, but was FFR was easier to breathe in compared to cloth
		FFR - nose trapped, couldn't breathe as easily, bad smell to mask.
		FFR - tight around nose, hard to breathe at times.
**Surgical**		
	Cloth mask was ok, surgical was slightly better.	Surgical mask moves around a lot and does not feel very helpful in filtering air supply.
The surgical mask was fine breathing		
I wouldn't choose to wear a mask but the surgical mask was definitely best.	Surgical was fine but sweaty.	The surgical mask accumulated a lot of water vapour and steamed my glasses.
The surgical mask was best- I could run with that.	Surgical mask was ok	Surgical mask moved around a lot on my face, uncomfortable.
Found FFR similar to the surgical mask - both were fine	Hospital mask was fine, bearable.	Didn't like surgical - steamed up glasses
No mask and surgical felt the same		
Surgical mask wasn't too bad, worked better with glasses than FFR.		
Found surgical mask fine.		
Surgical mask felt same as no mask don't really notice you're wearing it, although may think differently in the summer.		
There was little restriction with the surgical mask		
Hospital was best.		

For peer review only

Surgical mask was fine, easiest one.

For peer review only

Supplemental table 5. Participants written, free-text responses to the question, ‘Overall, how would you feel about being required to exercise wearing a face mask to limit the spread of COVID-19?’ These were categorised as being either supportive, against or neutral.

**Supportive**	**Against**	**Neutral**
If it was in the interests of public health and safety, I would have no issues wearing a mask to limit the spread of COVID-19. Of the masks worn, my strong preference would be for the FFP3 mask as it was by far the most comfortable	Do not feel it's worth the hassle	Was unpleasant exercising with any of the face coverings on but if I had to pick one to wear it would definitely be the surgical mask.
Would wear surgical mask if it was necessary	Not great. I would wear the surgical mask though	Mixed, the best mask was the respirator one but those are expensive to get. However, as an issue of public safety I wouldn't have a problem wearing one if told it was the best thing to do.
It would make no real impact to my exercise having to wear a face mask or not	Negative	It would depends on what type of mask I had. I would probably look into perhaps a running mask if such a thing exists. Failing that maybe a surgical one but it would certainly limit the exercise I would want to do as it is noticeably harder and less enjoyable to at least run in a mask.
It would be fine if it is moderate intensity exercise. If I was pushing extremely hard it may slightly limit me. If I was in a poorly ventilated gym I would feel more comfortable wearing a face mask	At least for exercise that significantly increase oxygen requirement, I would find it very challenging and would try to avoid	Not keen but would do it
Fine with it	I think its a really bad idea to have to wear a mask while exercising as it can make you very out of breath and I would fear it could even be fatal in some circumstances.	Fine but not ideal
Only would wear surgical mask	Wouldn't exercise	Wouldn’t mind if had to but would prefer not to
I would be happy to but it’s a lot less enjoyable	Would rather have a choice in the matter	Outside, I would not like it but it would be more pleasant as the wind is blowing on the rest of your face reducing the heat. Inside I would not like it due to the heat. It would definitely put me off exercising.
Fine if indoors and high risk setting but not so keen outdoors in large open spaces.	I would feel annoyed because outside its useless. It would make sense just inside	Surgical face masks would be a bit inconvenient but definitely an option especially in a gym context

For peer review only

I would be ok to exercise with the surgical mask, but none of the other ones	Would not support it at all. 2 out of 10	I would do what is necessary to protect others. However, with current evidence pointing towards the conclusion that isolated outdoors exercise would not effect COVID spread like eating in a pub would, I'm not inclined to wear one.
I think it is important to have a mask on when running in crowded public areas, otherwise it should not be required.	I believe we don't need to wear any face mask outdoors	If indoors - makes sense. Outdoors - a bit silly for me
Fine, just takes a while to adjust to wearing a face mask	I would still exercise but I wouldn't be happy. I'd also be annoyed because I don't think that it's really needed for preventing transmission, especially outdoors.	I would if I had to.
I would and have only used a surgical mask when required to wear one	I'd rather not unless I had to	I would rather not though if it was a rule I would do it to continue to exercise.
It is OK for me to wear a surgical mask (during exercise)	I would prefer not to	I'd wear the surgical mask if I had to wear something.
I agree to wear a face mask to do the exercise for safety, and the surgical mask did not interfere with my breathing	I'd prefer not to. If I had to I'd wear the surgical mask.	I would if needed to inside, don't see the point of having to wear one whilst exercising outside
I wouldnt mind if it was the surgical mask but it would definitely make me go slower	I wouldn't be happy with this, wearing a mask to exercise is uncomfortable and sweaty. When you're wearing one to exercise outdoor people give you looks, no one wears one outdoors	Would it have to, would choose the FFP
Exercising wearing a mask would be OK if we had to	Would prefer not to, if had to would wear FFR as didn't make glasses steam up	I wouldnt like to but would wear the surgical mask if we had to
Happy to wear the surgical face mask, but not the FFR or Cloth face mask	Would rather not, but would if really had to. Would choose to wear surgical.	I would tolerate it but not advocate it.
Wouldn't mind having to wear the surgical mask	Would rather not, would choose to wear surgical mask if had to, if forced to wear the others (cloth or FFR) I would choose not to exercise	
Felt it was possible to exercise in all 3, but less comfortable in FFR	Would prefer not to, wouldn't mind wearing surgical or cloth although not	

For peer review only

	ideal. Wouldn't wear FFR
I'd be happy to do it in an indoor gym if required to do so.	Wouldn't want to, hated it
	Wouldn't do it, would just go outside to exercise instead. Would consider hospital mask as that was the best.
I would be okay with it	I wouldn't be happy. When I ran with no mask at the end it felt like breathing in HD
Only if it was the surgical mask	
I would be fine with that.	
I would be happy to wear the surgical mask	
Having done so for the study, I wouldn't mind. Would choose to wear the surgical mask.	
Wouldn't mind doing so indoors, wouldn't mind wearing surgical or FFR	
Wouldn't mind doing so indoors, think it'd be unnecessary outdoors	
Wouldn't bother me too much, would choose to wear surgical.	
Not sure how they'd be in more intense exercise or hotter conditions. Would be fine on a run but more difficult if required to communicate in team sports e.g. football	
Would wear surgical mask, possibly buff for short distances were cardio is concerned	
Overall it was close to normal exercising wearing a mask. I would feel okay about doing this if it was not the cloth mask.	
To be honest if wearing a face mask during exercise will limit the spread of Covid then I am happy to do so	

For peer review only

Face masks and exercise (c29.9.20)

Page 1: Face masks and oxygen levels - what happens during exercise?

Introduction

Thank you for completing your exercise session, wearing the different face masks. We hope that you are now ready to upload your results. This should take around 10 minutes.

There are no right or wrong answers to any of the questions. Please be as honest as possible when answering. Your answers will be confidential and will not be shared with anyone other than the research team. We will not be collecting any identifiable information at this stage to protect your privacy.

By completing this questionnaire, you are consenting to your anonymous information being used for research on the impact of wearing a face mask whilst exercising.

Thank you again for supporting this research.

If you have any questions, please contact:

Dr Nicholas Jones (nicholas.jones2@phc.ox.ac.uk)

For peer review only - http://bmjopen.bmj.com/site/about/guidelines.xhtml

Page 2: Basic details

1. How old are you?

2. Which gender do you identify with?

3. How many days do you usually exercise per week?

4. Do you smoke?

4.a. If you smoke, how many cigarettes a day do you smoke on average?

5. What type of exercise did you do?

For peer review only - http://bmjopen.bmj.com/site/about/guidelines.xhtml

6. When did you complete the exercise session?

Please enter a date and time in the format 'DD/MM/YYYY HH:MM', for example 27/03/1980 15:43.

(dd/mm/yyyy hh:mm)

COVID screening questions

7. Where did you complete the study session?

- London
- Oxford
- Nottingham

8. Please confirm you do not have any symptoms of possible COVID-19 on the day of the study session

- No symptoms
- I have a fever
- I have a cough
- I have a change in sense of smell or taste

9. To the best of your knowledge, are other members of your household free from symptoms of COVID-19?

For peer review only - http://bmjopen.bmj.com/site/about/guidelines.xhtml

- ○ Yes
- ○ No

IF YOU OR SOMEONE YOU LIVE WITH HAVE ANY SYMPTOMS OF COVID-19, YOU MUST NOT TAKE PART IN THE STUDY TODAY. PLEASE LET A MEMBER OF THE STUDY TEAM KNOW AS SOON AS POSSIBLE.

For peer review only - http://bmjopen.bmj.com/site/about/guidelines.xhtml

Page 3: Oxygen saturations

The following questions will require you to input the oxygen saturation levels you recorded at different time points during each of the four exercise periods. Please also add your oxygen saturations at the start of exercise / resting oxygen saturations below.

10. What are your resting oxygen saturation levels?

11. Please record your oxygen saturations during exercise at each of the following time points. You should input an answer into every box.

	Oxygen levels 7 and a half minutes into exercise	Oxygen levels at the end of exercise	Oxygen levels 1 minute after ending exercise
No face mask	Please select	Please select	Please select
Cloth face mask	Please select	Please select	Please select
Surgical face mask	Please select	Please select	Please select
FFR (respirator) face mask	Please select	Please select	Please select

For peer review only - http://bmjopen.bmj.com/site/about/guidelines.xhtml

Page 4: Heart rate and exercise

The following questions will require you to input your heart rate levels, which you recorded at different time points during each of the four exercise periods. Please also add your heart rate at the start of exercise / resting heart rate below.

12. What is your resting heart rate?

13. Please record your heart rate during exercise at each of the following time points. You should input an answer into every box.

	Heart rate 7 and a half minutes into exercise	Heart rate at the end of exercise	Heart rate 1 minute after ending exercise
No face mask	Please select	Please select	Please select
Cloth face mask	Please select	Please select	Please select
Surgical face mask	Please select	Please select	Please select
FFR (respirator) face mask	Please select	Please select	Please select

For peer review only - http://bmjopen.bmj.com/site/about/guidelines.xhtml

Page 5: Distance travelled

Please record data here regarding the distance you travelled during exercise, including average speed data if available (please provide units).

14. Distance travelled exercising wearing **no face mask**

15. Average speed exercising with **no face mask**

16. Distance travelled exercising wearing a **cloth face mask**

17. Average speed exercising wearing a **cloth face mask**

18. Distance travelled exercising wearing a **surgical face mask**

For peer review only - http://bmjopen.bmj.com/site/about/guidelines.xhtml

19. Average speed exercising wearing a **surgical face mask**

20. Distance travelled exercising wearing a **FFR (respirator) face mask**

21. Average speed exercising wearing a FFR (respirator) face mask

For peer review only - http://bmjopen.bmj.com/site/about/guidelines.xhtml

Page 6: Comfort of wearing a face mask

22. How comfortable was the mask (or no mask) during this exercise session? (Rate from 1 to 9, 1= extremely comfortable, 9= extremely uncomfortable)

	Comfort level
No face mask	Please select
Cloth face mask	Please select
Surgical face mask	Please select
FFR (respirator) face mask	Please select

22.a. Were you able to complete all four exercises sessions? If not please state which you did not complete and why.

23. How easy did you find it to exercise during each session? (Rate from 0 to 10; 0=rest, 1=really easy, 2=easy, 3=moderate, 4=sort of hard, 5=hard, 7= really hard, 9= really, really hard, 10= maximal) [Borg Scale]

	Ease of exercise										
	0	1	2	3	4	5	6	7	8	9	10
No face mask	☐	☐	☐	☐	☐	☐	☐	☐	☐	☐	☐
Cloth face mask	☐	☐	☐	☐	☐	☐	☐	☐	☐	☐	☐
Surgical face mask	☐	☐	☐	☐	☐	☐	☐	☐	☐	☐	☐
FFR (respirator) mask	☐	☐	☐	☐	☐	☐	☐	☐	☐	☐	☐

24. Which face mask did you find most difficult to exercise wearing?

For peer review only - http://bmjopen.bmj.com/site/about/guidelines.xhtml

Please don't select more than 1 answer(s) per row.

	Cloth mask	Surgical mask	FFR (respirator) mask
Please select one	☐	☐	☐

25. How easy was it to breath during each exercise session? Please rate from 1 to 9, 1 = extremely easy, 9 = extremely difficult)

	1	2	3	4	5	6	7	8	9
No face mask	☐	☐	☐	☐	☐	☐	☐	☐	☐
Cloth face mask	☐	☐	☐	☐	☐	☐	☐	☐	☐
Surgical face mask	☐	☐	☐	☐	☐	☐	☐	☐	☐
FFR (respirator) face mask	☐	☐	☐	☐	☐	☐	☐	☐	☐

25.a. Please add any relevant comments you have on the ease of exercising wearing a face mask

26. Did you feel any other symptoms during exercise that were different to usual?

- ☐ Headache
- ☐ More breathless
- ☐ Claustrophobia
- ☐ Anxiety
- ☐ Fatigued
- ☐ Drowsy
- ☐ Dizzy
- ☐ Other

For peer review only - http://bmjopen.bmj.com/site/about/guidelines.xhtml

26.a. If other please specify

27. Overall, how would you feel about being required to exercise wearing a face mask to limit the spread of COVID-19?

For peer review only - http://bmjopen.bmj.com/site/about/guidelines.xhtml

CONSORT 2010 checklist of information to include when reporting a randomised trial*

Section/Topic	Item No	Checklist item	Reported on page No
**Title and abstract**			
	1a	Identification as a randomised trial in the title	1
	1b	Structured summary of trial design, methods, results, and conclusions (for specific guidance see CONSORT for abstracts)	2
**Introduction**			
Background and objectives	2a	Scientific background and explanation of rationale	4
	2b	Specific objectives or hypotheses	5
**Methods**			
Trial design	3a	Description of trial design (such as parallel, factorial) including allocation ratio	5
	3b	Important changes to methods after trial commencement (such as eligibility criteria), with reasons	6
Participants	4a	Eligibility criteria for participants	6
	4b	Settings and locations where the data were collected	6
Interventions	5	The interventions for each group with sufficient details to allow replication, including how and when they were actually administered	6-7
Outcomes	6a	Completely defined pre-specified primary and secondary outcome measures, including how and when they were assessed	6
	6b	Any changes to trial outcomes after the trial commenced, with reasons	N/A
Sample size	7a	How sample size was determined	6
	7b	When applicable, explanation of any interim analyses and stopping guidelines	N/A
Randomisation:			
Sequence generation	8a	Method used to generate the random allocation sequence	5
	8b	Type of randomisation; details of any restriction (such as blocking and block size)	5
Allocation concealment mechanism	9	Mechanism used to implement the random allocation sequence (such as sequentially numbered containers), describing any steps taken to conceal the sequence until interventions were assigned	5
Implementation	10	Who generated the random allocation sequence, who enrolled participants, and who assigned participants to interventions	5
	11a	If done, who was blinded after assignment to interventions (for example, participants, care providers, those	N/A

For peer review only - http://bmjopen.bmj.com/site/about/guidelines.xhtml

		assessing outcomes) and how	
	11b	If relevant, description of the similarity of interventions	4-5
	12a	Statistical methods used to compare groups for primary and secondary outcomes	7
	12b	Methods for additional analyses, such as subgroup analyses and adjusted analyses	7
	13a	For each group, the numbers of participants who were randomly assigned, received intended treatment, and were analysed for the primary outcome	7
	13b	For each group, losses and exclusions after randomisation, together with reasons	All volunteers enrolled
Recruitment	14a	Dates defining the periods of recruitment and follow-up	7
	14b	Why the trial ended or was stopped	Met sample size
Baseline data	15	A table showing baseline demographic and clinical characteristics for each group	Information provided in text, page 8
Numbers analysed	16	For each group, number of participants (denominator) included in each analysis and whether the analysis was by original assigned groups	Table 1, 2
Outcomes and estimation	17a	For each primary and secondary outcome, results for each group, and the estimated effect size and its precision (such as 95% confidence interval)	Table 1-4
	17b	For binary outcomes, presentation of both absolute and relative effect sizes is recommended	N/A
Ancillary analyses	18	Results of any other analyses performed, including subgroup analyses and adjusted analyses, distinguishing pre-specified from exploratory	Supplemental tables 1 to 3
Harms	19	All important harms or unintended effects in each group (for specific guidance see CONSORT for harms)	11
**Discussion**			
Limitations	20	Trial limitations, addressing sources of potential bias, imprecision, and, if relevant, multiplicity of analyses	14-15
Generalisability	21	Generalisability (external validity, applicability) of the trial findings	14
Interpretation	22	Interpretation consistent with results, balancing benefits and harms, and considering other relevant evidence	15-16
**Other information**			
Registration	23	Registration number and name of trial registry	17
Protocol	24	Where the full trial protocol can be accessed, if available	N/A
Funding	25	Sources of funding and other support (such as supply of drugs), role of funders	17-18

For peer review only - http://bmjopen.bmj.com/site/about/guidelines.xhtml

*We strongly recommend reading this statement in conjunction with the CONSORT 2010 Explanation and Elaboration for important clarifications on all the items. If relevant, we also recommend reading CONSORT extensions for cluster randomised trials, non-inferiority and equivalence trials, non-pharmacological treatments, herbal interventions, and pragmatic trials. Additional extensions are forthcoming: for those and for up to date references relevant to this checklist, see www.consort-statement.org.

For peer review only
